# Graph-based machine learning interprets and predicts diagnostic isomer-selective ion–molecule reactions in tandem mass spectrometry†

**DOI:** 10.1039/d0sc02530e

**Published:** 2020-10-05

**Authors:** Jonathan Fine, Judy Kuan-Yu Liu, Armen Beck, Kawthar Z. Alzarieni, Xin Ma, Victoria M. Boulos, Hilkka I. Kenttämaa, Gaurav Chopra

**Affiliations:** Department of Chemistry, Purdue University 560 Oval Drive West Lafayette IN USA hilkka@purdue.edu gchopra@purdue.edu; Purdue Institute for Drug Discovery, Integrative Data Science Institute, Purdue Center for Cancer Research, Purdue Institute for Inflammation, Immunology and Infectious Disease, Purdue Institute for Integrative Neuroscience West Lafayette IN USA

## Abstract

Diagnostic ion–molecule reactions employed in tandem mass spectrometry experiments can frequently be used to differentiate between isomeric compounds unlike the popular collision-activated dissociation methodology. Selected neutral reagents, such as 2-methoxypropene (MOP), are introduced into an ion trap mass spectrometer where they react with protonated analytes to yield product ions that are diagnostic for the functional groups present in the analytes. However, the understanding and interpretation of the mass spectra obtained can be challenging and time-consuming. Here, we introduce the first bootstrapped decision tree model trained on 36 known ion–molecule reactions with MOP. It uses the graph-based connectivity of analytes' functional groups as input to predict whether the protonated analyte will undergo a diagnostic reaction with MOP. A Cohen kappa statistic of 0.70 was achieved with a blind test set, suggesting substantial inter-model reliability on limited training data. Prospective diagnostic product predictions were experimentally tested for 13 previously unpublished analytes. We introduce chemical reactivity flowcharts to facilitate chemical interpretation of the decisions made by the machine learning method that will be useful to understand and interpret the mass spectra for chemical reactivity.

## Abstract 2

We combine mass spectrometry with machine learning that is predictive and explainable using chemical reactivity flowcharts for diagnostic ion–molecule reactions.

## Graphical abstract

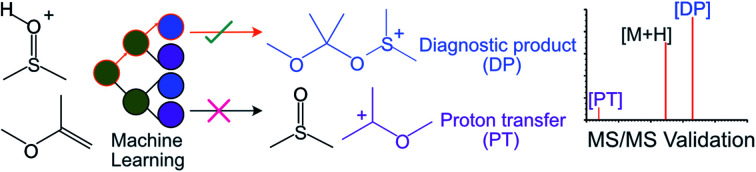

## Introduction

Tandem mass spectrometry (MS/MS) is a powerful analytical tool that is extensively used for the characterization of complex mixtures in many fields, such as proteomics, petroleomics, and drug discovery.^1–4^ Currently, the most commonly used MS/MS technique to obtain structural information for ionized and isolated mixture components is collision-activated dissociation (CAD).^5,6^ In these experiments, the analyte ions are accelerated and allowed to collide with an inert gas, such as helium. Upon the collisions, part of the kinetic energy of the ions is converted into their internal energy, resulting in fragmentation. This approach is limited by the fact that isomeric ions often generate identical fragmentation patterns, making identification of compounds *via* CAD mass spectra unreliable.^4,7^ To address this issue, a MS/MS approach based on diagnostic, reliable and predictable gas-phase ion–molecule reactions has been developed.^7–11^ This approach can be used to identify specific functional groups or their combinations in ionized and isolated mixture components to thereby facilitate the differentiation of isomeric ions, often without the need for reference compounds. No specialized instrumentation is needed for these experiments. The only modification to any commercial ion trap or multiquadrupole instrument is the addition of an inlet system for the neutral reagents, which is straightforward.^7,8,12,13^

**Fig. 1:**
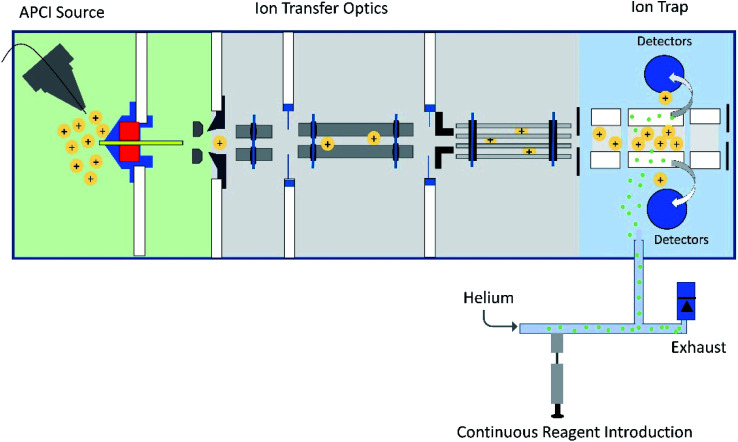
Schematic diagram of a linear quadrupole ion trap mass spectrometer equipped with an APCI source and an external reagent mixing manifold (bottom).^12,13^ This instrument can be used to detect diagnostic ions formed between analytes protonated upon APCI and a neutral reagent (introduced using the reagent mixing manifold) in MS/MS experiments occurring in the ion trap.

**Fig. 2:**
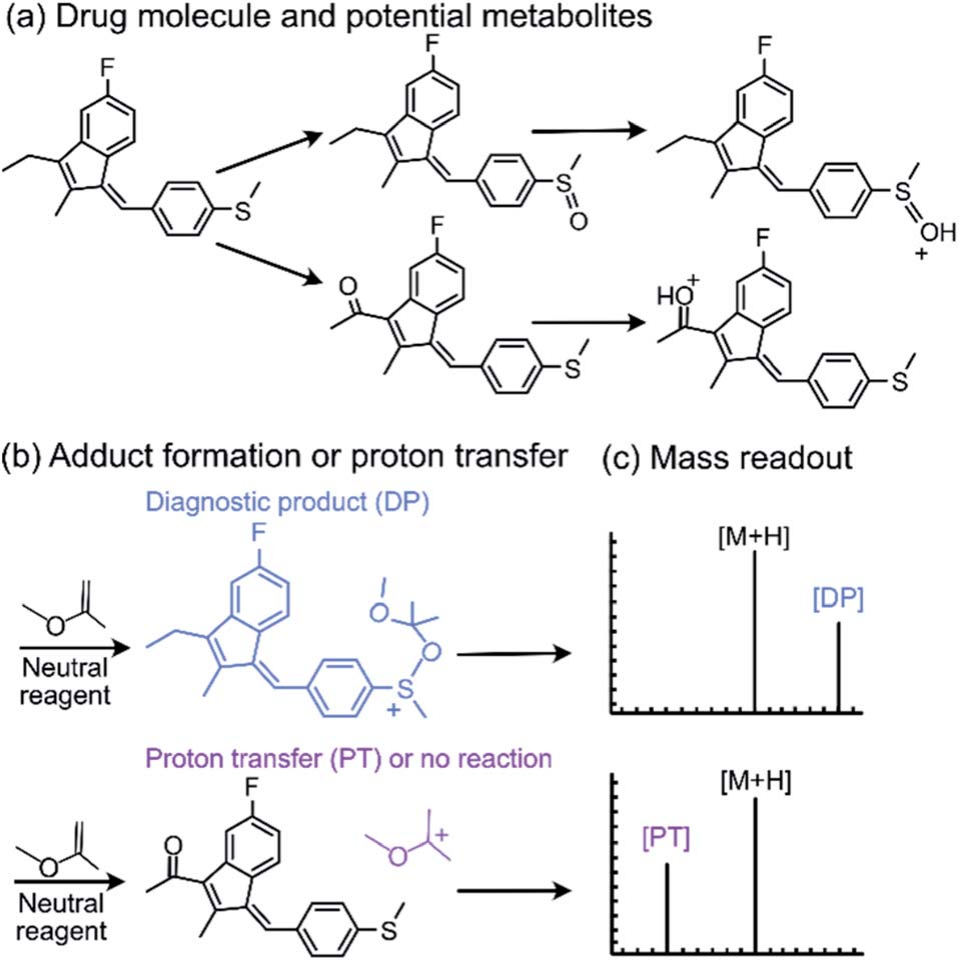
The diagnostic utility of employing neutral reagents, such as MOP, to identify functional groups in protonated metabolites of a drug. After the metabolites were (a) protonated and isolated, (b) they were allowed to react with MOP and (c) the formation of a diagnostic addition product (DP) as opposed to proton transfer (PT) or no reaction was monitored. Only the protonated sulfoxide metabolites generated the diagnostic addition product ion (DP) with MOP.

One of the neutral reagents used previously to differentiate two isomeric drug metabolites is 2-methoxypropene (MOP).^7^ In these experiments, protonation of the analytes was achieved through atmospheric pressure chemical ionization (APCI) in a linear quadrupole ion trap mass spectrometer. The protonated analytes were transferred into the ion trap, isolated and allowed to react with MOP that was continuously introduced into the ion trap (Fig. 1). Some protonated analytes were unreactive toward MOP and others transferred a proton to MOP. The protonated analytes of the greatest interest here are those that formed a diagnostic, stable addition product with MOP. All generated product ions were ejected in a mass-selective manner from the ion trap into external detectors to determine their *m*/*z*-values and relative abundances. This enabled the determination of reactions that had taken place. The product branching ratios (defined as the abundance of a stable adduct divided by the abundances of all product ions) were measured at several reaction times and were constant over time. The diagnostic addition product ions were only observed for the protonated sulfoxide drug metabolite and not for its keto-isomer (Fig. 2). This was verified *via* studies of several protonated model compounds.^8^

Interpretation of the data obtained for complex mixtures in the above experiments is challenging and time-consuming due to the large amount of the data. Therefore, we decided to develop a chemical graph based interpretable machine learning methodology to facilitate data interpretation and the prediction of whether a given protonated analyte will form a diagnostic product ion upon reactions with MOP. Previously, multilayer-perceptron,^14–16^ Long-Short Term Memory^17,18^ (LSTM) and Graph Convolution Networks^19–22^ (GCN) approaches have been demonstrated to be suitable for predicting reaction outcomes when a large number of known reactions are available. Unfortunately, due to the specificity of the diagnostic ion–molecule reactions of interest here, only a relatively small set of known reactions exist. Furthermore, these models are difficult to understand and yield no additional chemical insight. Although one-shot and few-shot learning has proven useful in the literature for systems with a small number of observations,^23–26^ these models are typically difficult to interpret and only limited information can be obtained about the reactions. Therefore, a machine learning methodology that can be interpreted by humans is developed in this work and termed a “chemical reactivity flowchart.”

Previously, the proton affinity (PA) of an analyte was used to predict whether a protonated analyte would undergo diagnostic product formation, proton transfer, or no reaction with MOP.^8^ If the PA of the analyte is lower than that of MOP, proton transfer usually dominates. On the other hand, if the PA of the analyte is greater than that of MOP, a diagnostic adduct may be formed. However, accurate predictions between formation of the diagnostic adduct and no reactions were not possible. Nevertheless, PA values may be used as a baseline for benchmarking potential machine learning methods or as a source for additional input features.

## Results and discussion

### Choice of the machine learning model

Given the sparsity of data available for training a machine learning model, traditional architectures known to perform well with small amounts of data were evaluated. These machine learning architectures include regularized logistic regression,^27,28^ decision tree models,^29,30^ partial least squares,^31^ generalized linear models,^32^ and *k*-nearest neighbor.^33^ Each of these models solves classification problems in a very different manner. For example, logistic regression attempts to assign numeric weights to an input vector this vector is then used to linearly transform the input into two probabilities for assignment of the input as a given class. On the other hand, decision trees (when trained for classification) attempt to reduce the Shannon entropy of the predicted class by splitting the data using a set of Boolean operations. This yields a flowchart of logical decisions that one can use to evaluate the decisions made by the model (see the Methods section for details of this procedure). The major advantage of decision tree models, with analytes represented as an input bit vector of functional groups, is that the resulting flow chart diagram can be interpreted by chemists to gain a deeper understanding of the chemistry resulting in a reaction taking place. This procedure is widely used in both biology^30^ and chemistry^34,35^ to identify and interpret how input features (in this case the collection of functional groups) correlate with a property of interest (reactivity toward MOP in this case). Recently, similar techniques have been applied to reaction chemistry^36^ to understand how various chemical moieties are related to the reactivity of a molecule. Here, we used bootstrapping of several decision tree models to ensure robustness of our model for prospective experimental validations. Moreover, a comparison of the performance of decision trees to other machine learning models was also performed to ensure that efficacy was not compromised for the sole sake of interpretability.

To develop a chemically interpretable machine learning model, the presence or lack of a topology of a collection of atoms (referred to as functional groups) was related to predicted reactivity. The Morgan fingerprint algorithm^37,38^ was used to represent such functional groups, avoiding the use of manually created functional groups subject to human bias and interpretation. Additionally, previous work indicates that the use of Morgan fingerprints in machine learning is an effective approach across chemical disciplines.^39–41^ Briefly, this algorithm functions by finding all subgraphs of a molecular graph (*i.e.* the connectivity of the atoms in the molecule) and assigns a number to these subgraphs calculated *via* a set of hashing functions applied to each atom and its respective neighborhood. This yields an integer which can be used as a surrogate for the functional group. The size of these subgraphs was determined by a radius parameter that is supplied by the user *a priori*. Application of a small radius in machine learning has been shown to avoid the potential for the same integer to represent the same functional group, a phenomenon known as a bit collision.^42^ In this work, the ability of models trained on different radii were also compared to ensure that the selection of fingerprint radius is optimal for the task at hand.

### Cutoff assignments for the machine learning model

**Fig. 3:**
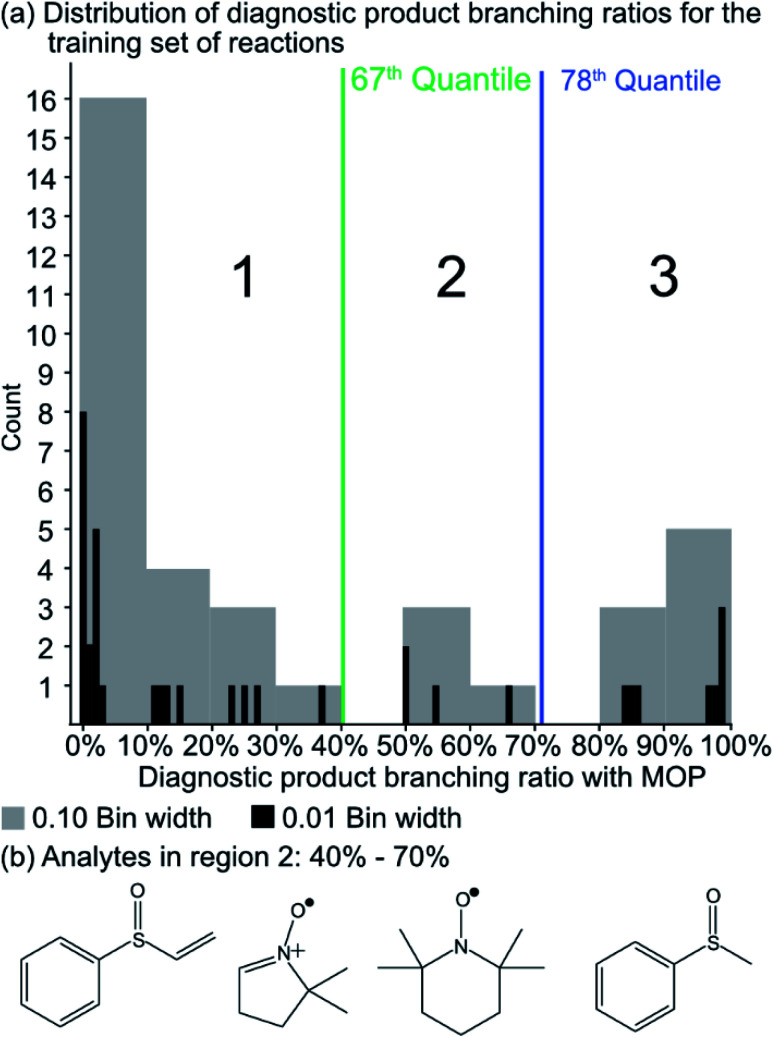
(a) The distribution of diagnostic product branching ratios for the initial training set of 36 reactions. (b) Structures for representative analytes with diagnostic product branching ratios between 40 and 70%.

Since the experimental outcome of a given analysis was either proton transfer/no reaction, or the formation of a diagnostic addition product ion (see Fig. 2), and a limited amount of data were available for training, a binary classifier is preferable to other supervised machine learning models. The training set for this classifier included a set of 36 protonated analytes whose reactions with MOP have been studied along with their product branching ratios^8,43,44^ (see Table S1† for all MOP reactions). The distribution of product branching ratios measured for the diagnostic addition reaction (see Fig. 3a) shows a large gap between 65–83% as no compounds have a diagnostic product branching ratio between this percentage gap. This gap indicates that a cutoff of 70% or greater for the branching ratio should be used in this binary classifier to determine whether a given analyte will undergo the diagnostic addition reaction with MOP.

The selection of the above cutoff resulted in 8 protonated analytes being classified as forming a diagnostic addition product ion with MOP and 28 protonated analytes being considered as non-diagnostic. Since this split was unbalanced (*i.e.* more nondiagnostic reactions than diagnostic), the Cohen kappa statistic^45^ was used to compare the success of different models. A kappa statistic of zero indicates that the model performs at random and a value of positive 1 indicates a perfect classifier (see Methods section for details). To further investigate the effects of this cutoff value, models created with a 70% cutoff were compared to those created with 10%, 20%, 30%, 40%, 50%, 60%, and 90% cutoffs to ensure that this choice was logical with respect to how the models performed for reactions not used to train the model. Note that a cutoff of 80% was not considered as it produced the same set of analytes that underwent the diagnostic reaction as the 70% cutoff.

A potential alternative to the 70% cutoff is 40% as this represents the second-largest gap in the distribution of diagnostic product branching ratios (see Fig. 3a). This value is approximately at the 67^th^ quantile of the data and resulted in a split of 13 analytes that underwent the diagnostic addition reaction, compared to 23 analytes that did not. When considering the result of the binary classifier with different cutoffs, 70% and 40%, the model classified four analytes, TEMPO (an *N*-oxide radical), 5,5-dimethyl-1-pyrroline *N*-oxide, methyl phenyl sulfoxide, and (ethenesulfinyl)benzene (a sulfoxide) (see Fig. 3b), differently. Conversely, with both cutoffs, the model classified all sulfones, alcohols, and amines to undergo proton transfer or no reaction instead of forming a diagnostic addition product ion. The similarities and differences between the 70% and 40% cutoffs could be used to further understand how the model performs and assigns classifications.

To ensure that a decision tree model will perform well prospectively, 13 compounds that were not present in the training set (*i.e.*, test set) were evaluated using a bootstrapped set of models trained with different diagnostic branching ratio cutoffs. In addition, models were trained using different fingerprint radii to ensure that a radius of 1 is appropriate (see ESI† for details). These 13 compounds (Table 1) were selected from an in-house library of available compounds and the model was prospectively tested using ion–molecule reactions with MOP. These 13 compounds were selected based on a criterion that either their functional groups were not present in the compounds of the training set or all bootstrapped decision tree models resulted in the prediction of formation of a diagnostic addition product with MOP. The results are shown in Table S2 and Fig. S2–S14.†

**Table 1:** The probability for assignment of a correct reaction for all decision tree models

#	Test compound	Formation of diagnostic product^a^	20%	30%	40%	50%	60%	70%	Proton affinity (kcal mol^−1^)
1	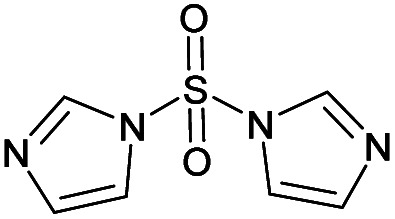	Yes	51%	54%	50%	47%	100%	100%	214.43^b^
2	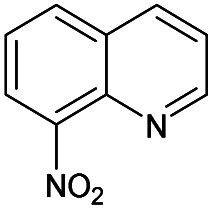	No	0%	8%	0%	0%	0%	0%	225.23^b^
3	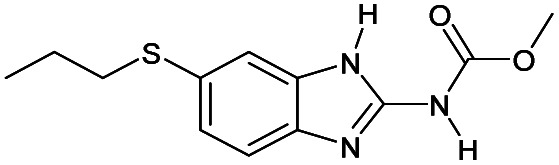	No	0%	8%	0%	0%	33%	0%	229.51^b^
4	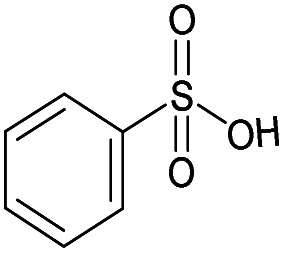	No	0%	0%	0%	0%	0%	0%	188.57
5	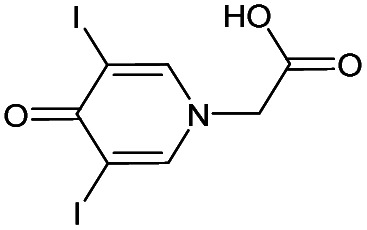	No	59%	58%	50%	44%	4%	0%	222.71^b^
6	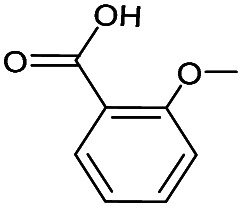	No	0%	0%	0%	0%	33%	0%	195.01
7	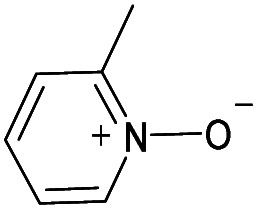	Yes	100%	100%	100%	94%	100%	100%	224.15^b^
8	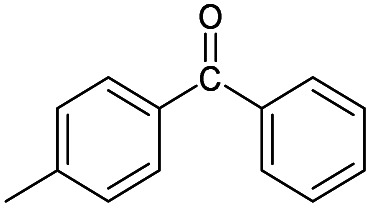	No	0%	0%	0%	0%	33%	0%	214.36
9	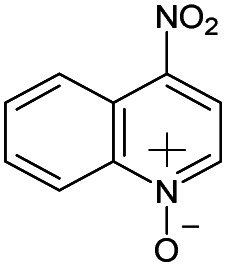	No	100%	100%	100%	100%	100%	100%	213.07
10	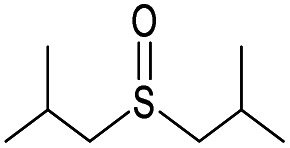	Yes	100%	100%	100%	100%	61%	100%	228.46^b^
11	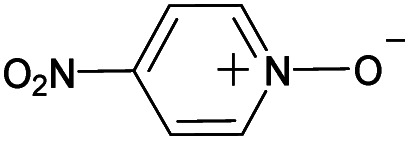	No	100%	100%	100%	94%	100%	100%	205.64
12	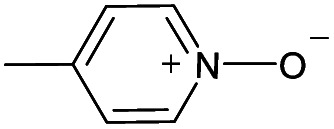	Yes	100%	100%	100%	88%	100%	100%	226.38^b^
13	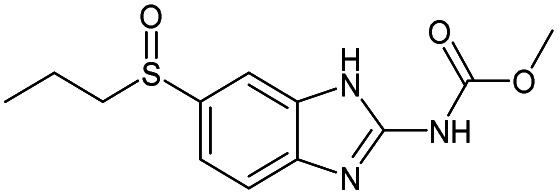	Yes	100%	100%	100%	100%	100%	100%	232.58^b^
	Kappa value	—	0.56	0.56	0.56	0.53	0.70	0.70	0.40

a See Fig. S2–S14 for assignment of diagnostic production formation.

b Value greater than the proton affinity of MOP (214.42 kcal mol^−1^) as calculated using density functional theory, see section Calculation of proton affinities in Methods for details.

The probabilities of the analytes to form a diagnostic product as assigned by the radius 1 decision tree models are given in Table 1 and for other radii in Table S3.† These tables show that the 60% and 70% cutoffs produced the models best suited for the external test set with a kappa value (0.70) that is greater than for the other cutoff values. The prediction probabilities for the analytes that underwent no diagnostic reaction (#3, #5, #6, and #8) were zero in the 70% cutoff model but above 30% in the 60% cutoff model. Therefore, the 70% cutoff was superior to 60% as it produced lower probabilities of diagnostic addition product formation for analytes that predominantly reacted *via* proton transfer or not at all. Additionally, other machine learning methods, including regularized logistic regression, *k*-nearest neighbor, and partial least squares classification (Tables S4–S7†), were evaluated. None of these methods outperformed the 70% decision tree model trained with a fingerprint radius of 1. Finally, the proton affinity model achieved a kappa value of 0.40, indicating that the decision tree model significantly outperformed the manual approach of identifying reactions based on proton affinities. One should note that the proton affinities relevant to test reactions #1 and #8 and the calculated proton affinity of MOP are all within 0.1 kcal mol^−1^ of each other. Therefore, the correct ordering of these proton affinity values may not have real significance. Moreover, using the experimental value for the MOP proton affinity instead of the calculated value results in a kappa value of 0.43, further demonstrating the superiority of the decision tree model (kappa = 0.70) over that of proton affinity calculations.

**Fig. 4:**
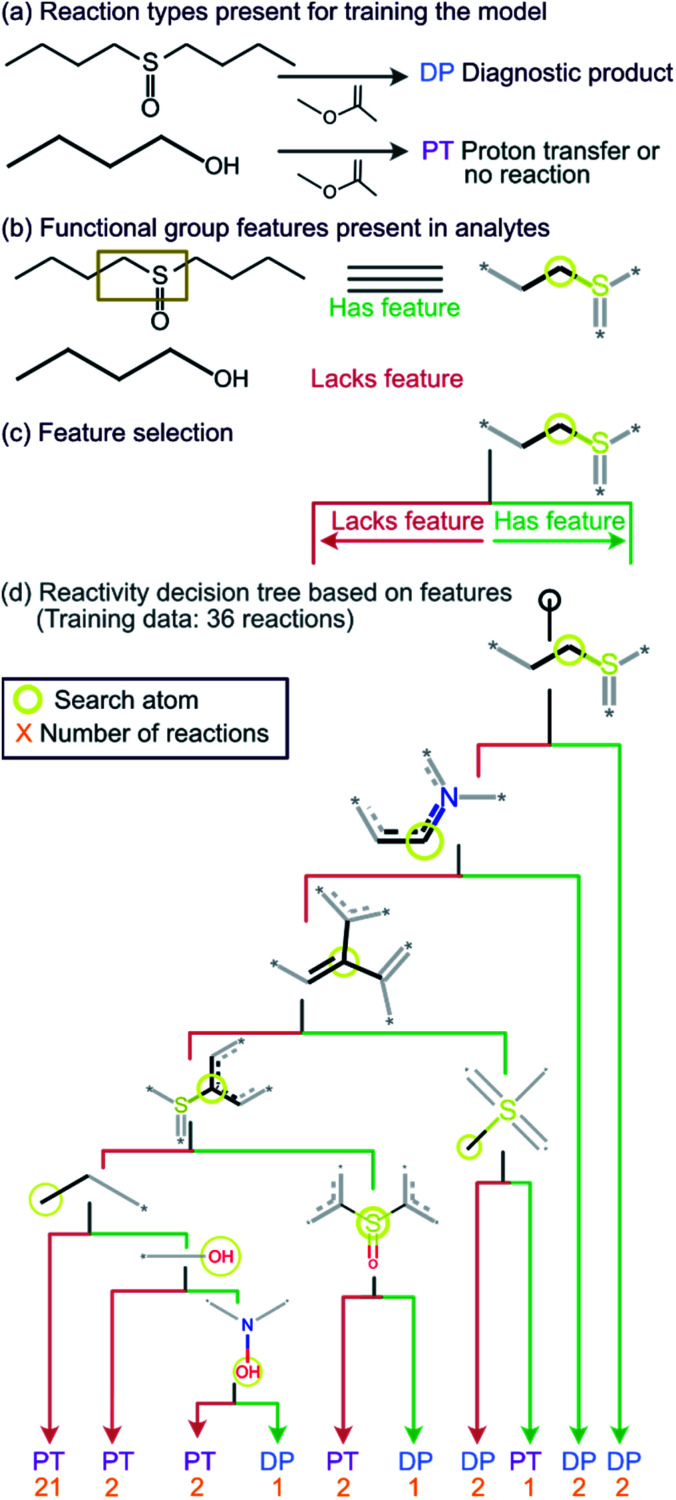
Chemical reactivity flowchart. (a) Analytes that form the diagnostic product (DP) or undergo proton transfer or no reaction (PT). (b) Compounds identified as having a specific functional group feature (left), such as a sulfoxide with at least one aliphatic carbon atom bound to it (right). No structure is shown when the feature (sulfoxide) is absent in the molecule that does not form a DP. (c) Flowchart for decision making based on the presence or absence of the feature (sulfoxide). (d) The decision tree model trained on a diagnostic product branching ratio cutoff of 70%. The model classifies analytes as reactive or unreactive towards MOP based on their functional groups determined by the Morgan algorithm with a radius of 1 atom.

Given the straightforward interpretability of decision tree models, we introduce a chemical reactivity flowchart to rationalize the logic behind the 70% model used here to make predictions. The decision tree flow chart for the 70% cutoff and the fingerprint radius of 1 atom is given in Fig. 4 and a chart for the 40% cutoff is provided in Fig. S1 (ESI†). The logic begins by checking for the presence of a sulfoxide functionality with at least one aliphatic carbon atom bound to it in the analyte and, if found, the analyte is assigned as “reactive” (see Fig. 4d). Then, the model checks for the presence of a nitrogen atom with three substituents in a heteroaromatic ring (note that dashed lines indicate an aromatic bond) and assigns the analyte as “reactive” if such an atom is present. If neither functional group is present, the model checks for a junction between sp^2^ hybridized atoms and assigns analyte containing this group as “reactive”. If this group is not present, the model checks for a sulfoxide group located next to one or more aromatic rings and assigns the analyte as “reactive” if the sulfoxide group is between two aromatic rings. After this, the model checks for a terminal carbon bound to any atom and assigns all analytes lacking this functionality as “unreactive”. Those analytes that contain this functionality are checked for terminal oxygens or carbonyl groups and compounds lacking these functionalities are checked for a hydroxylamino group for final “reactivity” assignment. It should be noted that these features are identified by the trained decision tree model and that they make chemical sense in several cases, such as that compounds containing sulfoxide group with at least one aliphatic carbon atom bound to it (feature) generating the diagnostic product with MOP (Fig. 4).

All cutoff models correctly predicted the two test sulfoxide compounds (#10 and #13 in Table 1) to be “reactive” towards MOP. This result can be explained by the fact that all protonated sulfoxides in the training set except for one (tetrahydrothiophene 1-oxide, analyte #22 in Table S1 in ESI†) had a reaction efficiency greater than 40%. Therefore, the model predictions reflect the true experimental conclusion regarding sulfoxide compounds. This concept was reflected by the presence of a sulfoxide group as the paramount feature in the model (at the top of Fig. 4a–c). Similarly, all the models predicted that analytes containing an *N*-oxide functionality are “reactive” (#7, #9, #11, and #12). However, experimental results show that compounds containing nitro groups (#11 and #9) are “unreactive” (do not undergo a diagnostic addition reaction). These two compounds represented the only two errors made by the 70% cutoff model and these failures may be due to a nitro group not being present in compounds in the training set. The proton affinity model, however, correctly predicted these two nitro compounds as “unreactive” towards MOP, suggesting that when new functional groups are added into the model, a proton affinity verification step could be used to ensure that the new reaction predictions are correct. Since proton affinity model incorrectly predicted that compounds #2, #3, and #5 will form diagnostic addition products and that compound #1 will not, and none of these compounds contain functional groups present in the training set, it is best to apply this verification only if the compound contains functional groups not present in compounds in the original training set.

### Retraining the decision tree model on new reactions

**Fig. 5:**
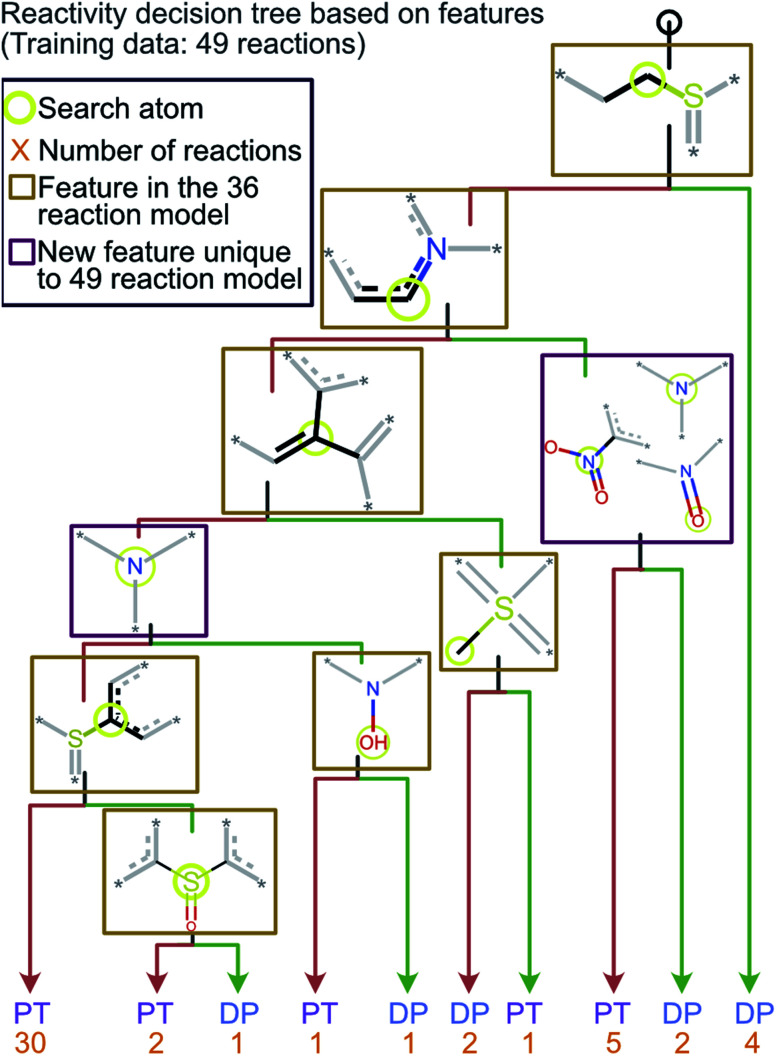
The decision tree model obtained by retraining the first model by using the 70% cutoff and all 49 reactions (original 36 and new 13 test reactions). This model is similar to the one obtained *via* a training set of 36 reactions but has an additional check for a nitro group which was not included in the original model. The lack of any major changes from the model shown in Fig. 4 indicates that the final model is robust and is able to incorporate new functional groups.

To ensure that the introduction of new data does not cause extensive changes to the decision tree model, a new model was trained with the addition of 13 analytes to the initial 36 analytes in the training set. The new model obtained by training with all these 49 analytes is shown in Fig. 5. The minimal changes in the chemical features seen in this model indicate that the new model does not have many logical changes as compared to the previous model shown in Fig. 4. The first three comparisons were the same between both the original 36-analyte model and the new 49-analyte model and the new model only introduced four additional functional groups. Three of these new functional groups were related to the nitro group present in the compounds in the new training set: 4-nitropyridine *N*-oxide and 4-nitroquinoline *N*-oxide. Therefore, one can deduce that the model has added an additional comparison to prevent these compounds from being predicted as “reactive”. As more protonated analytes with known reactivities towards MOP are identified, this model can be retrained to incorporate these new analytes, yielding improved predictions in the future while retaining baseline performance and simplicity.

## Conclusions

The work presented here demonstrated that a combination of machine learning and tandem mass spectrometry experiments based on diagnostic ion–molecule reactions can be used to identify or rule out specific functional groups in protonated analytes in a semiautomated fashion while generating results in a manner readily understandable to chemists. When many diagnostic reactions are examined, and this information is combined with additional information obtained from other mass spectrometry or spectroscopy evidence, identification of the analyte is facilitated. This machine learning methodology combined with an automated functional group identification method (Morgan fingerprinting) with a decision tree model trained on only 36 analytes and was prospectively validated using 13 external analytes of unknown experimental outcomes. The model correctly predicted reactivity for 11 of the 13 analytes present in the test set without any additional proton affinity-based QM calculations, and 13 of 13 analytes when an additional QM filter based on the relevant proton affinities was applied. In addition to outperforming other traditional machine learning models, the decision tree model is easily interpretable by humans using the chemical reactivity flowcharts shown in this work. Additionally, the inclusion of new data resulted in only minor changes to the model as opposed to the creation of an entirely new model, which suggests a robust selection of chemical features.

Over the years, more than 20 different reagents have been developed, with more developed every year, for the identification of different functionalities in protonated analytes.^4,7–9,11,13,43,44^ Chemical diversity in the reagents results in increased reliability of the identification. At this time, nine different reagents can be tested simultaneously by using a nine-pulsed valve system developed for rapid introduction of nine reagents while each analyte in a mixture elutes from an HPLC column.^46^ The experiment is automated. Therefore, nine reagents and many analytes can be tested in one HPLC run. Integration with interpretable machine learning methods will improve the decisions for the selection of nine reagents for subsequent HPLC runs towards development of autonomous systems.

While the integration of machine learning methods to gas-phase ion–molecule reactions and data analysis is not widely adopted nor commercially available currently, it is likely to become much more common in the future when its full power has been demonstrated. The methodologies presented herein will pave the way for expanding the above MS/MS method to include new diagnostic reactions for the identification of many different functionalities in, for example, drug metabolites in an easy, accurate, and automated manner. The ultimate goal of this research is to develop methodology for the fast determination of unknown isomeric metabolites of medicinal compounds *via* the identification of diagnostic product ions formed with selected neutral reagents. In the future, a fully automated pipeline for mixture component identification incorporating multiple models similar to the one presented here will be showcased along with how this methodology can be used to aid in the development of new therapeutics. The detailed output of all machine learning models is given in the ESI† along with the MS/MS spectra measured for all MOP reactions not previously reported in the literature. Additionally, all computer code, machine learning inputs, and other relevant scripts are provided on our GitHub page: https://www.github.com/chopralab/mop_reactivity_analysis.

## Methods

### Safety statement

No specific safety protocols were required for this research as all none of the analytical techniques used resulted in unsafe chemical environments.

### Materials

All chemicals were purchased from Sigma Aldrich and used without further purification.

### Mass spectrometry

All experiments were performed using a Thermo Scientific linear quadrupole ion trap mass spectrometer (LQIT) equipped with an atmospheric pressure chemical ionization (APCI) source and operated in positive ion mode. Sample solutions were prepared at concentrations ranging from 0.01 to 1 mg mL^−1^ with methanol as the solvent. The solutions were injected into the APCI source through a syringe pump at a rate of 15 μL min^−1^ by using a 500 μL Hamilton syringe. In the APCI source, typical flow rates for sheath and auxiliary gases (N_2_) were 30 and 10 (arbitrary units), respectively. The vaporizer and capillary temperatures were 300 and 275 °C, respectively. The ions generated upon APCI were transferred into the ion trap. The voltages applied to the ion optics were optimized for each protonated analyte *via* the tune feature of the LTQ Tune Plus interface. The neutral reagent, MOP, was introduced into helium buffer gas line of an external reagent mixing manifold *via* a syringe pump operating at a rate of 5 μL h^−1^.^12,13^ The surrounding areas of the syringe port were heated to about 120 °C to ensure that MOP evaporated completely. MOP was then diluted and directed into the ion trap by a constant flow of helium gas, controlled by a leak valve. Protonated analytes were isolated using an isolation width of 2 *m*/*z* units and a *q* value of 0.25, and then allowed to react with MOP in the ion trap for up to 10 000 ms. After this, all ions were detected using external electron multipliers. The MS/MS spectra measured for the new analytes studied in this paper are given in Fig. S2–S14† for one reaction time The diagnostic product branching ratio is the fraction of product ions that have the mass of the analyte plus a proton plus the mass of MOP relative to all product ions generated. These values were measured at several reaction times and found to be constant with time as no secondary reactions took place. The reproducibility of these measurements was found to be better than ±10%. Other potential product ions are MOP + proton and the diagnostic adduct minus the mass of methanol.^8,43,44^

### Creation and evaluation of the decision tree models

The prediction of adduct formation of a protonated analyte with MOP is possible through a combination of fingerprinting techniques and corresponding machine learning techniques. For each reaction, the protonated analyte and adduct were written as a stoichiometrically-balanced reaction-SMILES string. The field for the ‘name’ of the reaction is annotated with the diagnostic product ratio as shown in Table S1.† This reaction was then converted to a Morgan fingerprint^37^ using the RDkit software package^47^ with a radius of one, two, and three atoms and a bit length of 2048 bits. For the sample case presented herein, 36 reactions (training set) of known protonated analytes with the MOP reagent were examined^8,42,43^ in the decision tree model and each reaction was assigned a binary response of “no-hit” or “hit” based on the branching ratios of the products. The decision tree models were created using, the Julia implementation of Decision Tree, DecisionTree.jl (https://github.com/bensadeghi/DecisionTree.jl) using a minimum leaf size of 2 to reduce overfitting to a single analyte. The decision tree classification model increases the information gain (IG) of a given classification (*c*, in this case simply “no-hit” or “hit”) by decreasing the entropy (*H*) of a given set (*S*) which was calculated using eqn (1) using the probability of finding a specific class (*p*(*c*)). Information gain is the difference in entropy for the full set (*S*) and the sum of the entropies for all subsets (*t*) created by all splits (*A*) as shown in eqn (2).^29^1
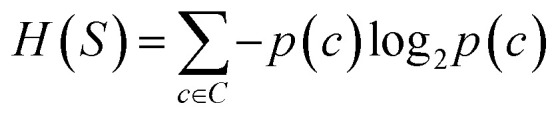
2
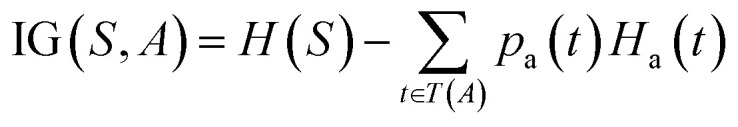


A bootstrapping technique was used to address the fact that the creation of an individual decision tree model relies on the selection of random input features to be used as splits. Through this technique, 10 000 decision tree models were created for each radius and cutoff value and the frequency of each functional group used by the models was measured along with the number of times a given test analyte was predicted to be “reactive” toward MOP. The frequencies of the functional groups were used to create the chemical reactivity flowcharts shown in Fig. 4, 5 and S1.†

For the logistic regression,^27,28^ partial least squares,^31^ generalized linear models,^32^ and *k*-nearest neighbor predictions,^33^ the Caret software package^48^ was utilized to create and evaluate the models. A simple grid search was performed to obtain a set of optimal hyperparameters. The input features were the Morgan fingerprint bit-vectors and the output was the binary outcome of whether the protonated analyte would be “reactive” toward MOP.

### Calculation of the kappa statistic

The kappa statistic^45^ was calculated using the following terms. True positive (TP) is the number of analytes predicted to be reactive that were experimentally found to be reactive while false positive (FP) is the number of analytes predicted to be reactive but that were experimentally not found to be reactive. Similarly, true negative (TN) is the number of analytes predicted to be unreactive that were experimentally found to be unreactive and false-negative (FN) is the number of analytes predicted to be unreactive that were experimentally found to be reactive. Based on these definitions, accuracy (*P*_o_) is defined using eqn (3).3
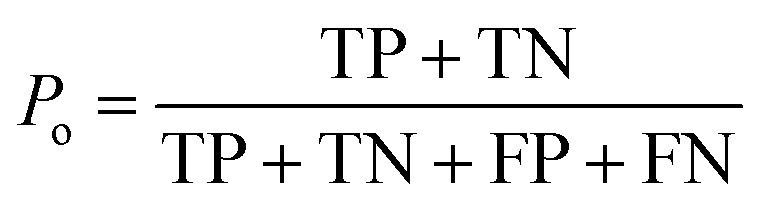


Additionally, the chance probability (*P*_e_) of the model defined to predict the reactivity correctly was determined using eqn (4).4
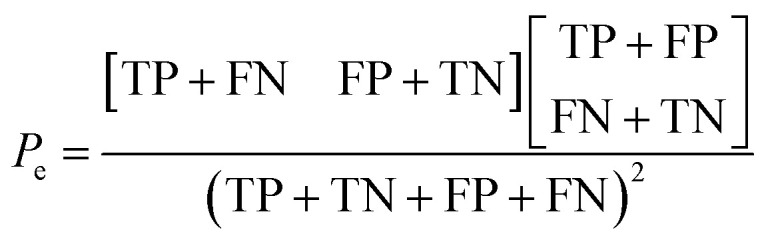


Finally, the kappa statistic was calculated using eqn (5).5
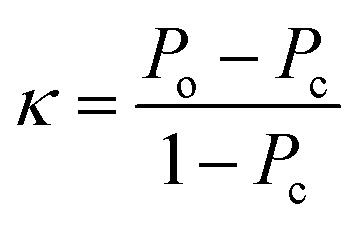


### Calculation of proton affinities

All quantum chemical calculations were performed using Gaussian 16 revision B.01 (ref. 49) and the M06-2x density functional.^50^ The 6-311++G(d,p) basis set was employed for all compounds except for 3,5-diiodo-4-pyridone-1-acetic acid that was calculated using the D-Gauss Double Zeta Valence Polarized basis-set (DGDZVP) to account for the iodine atoms.^51^ The three-dimensional structures for all analytes were constructed using the ‘Clean Structure in 3D’ feature as implemented in MarvinSketch.^52^ Then, GaussView^53^ was used to add protons to generate protonated molecules (see Table S2† for the location of the additional proton). The resulting structures were optimized and the difference between the electronic energies for the neutral and the protonated molecules was determined and compared to the known proton affinity of a simple reference compound used in an isodesmic reaction. Here, methanol was used when the proton affinity was calculated for an oxygen atom, ammonia was used when the proton affinity was calculated for a nitrogen atom, and 2-methyl propene is used for MOP. See Table S2† for individual proton affinity values and the associated content on https://www.github.com/chopralab/mop_reactivity_analysis for the Gaussian 16 input and output files respective to the aforementioned calculations.

## Conflicts of interest

The authors declare no competing financial interests.

## Supplementary Material

SC-011-D0SC02530E-s001
