# Supplementary material for: Graph-based machine learning interprets and predicts diagnostic isomer-selective ion–molecule reactions in tandem mass spectrometry
Source: Chem Sci. 2020 Oct 5;11(43):11849–58. doi: 10.1039/d0sc02530e (PMC8162943; doi:10.1039/d0sc02530e)
Supplement: SC-011-D0SC02530E-s001 [file SC-011-D0SC02530E-s001.pdf]

Electronic Supplementary Material (ESI) for Chemical Science.
This journal is © The Royal Society of Chemistry 2020

SUPPORTING INFORMATION

Graph-based machine learning interprets and predicts diagnostic isomer-selective ion-molecule reactions in tandem mass spectrometry

Jonathan Fine,[#,1] Judy Kuan-Yu Liu,[#,1] Armen Beck,[1] Kawthar Z. Alzarieni,[1] Xin Ma,[1] Victoria Boulos,[1] Hilkka I. Kenttämaa,[*,1] Gaurav Chopra[*,1,2]

[#] These authors share an equal contribution to this work

[1] Department of Chemistry, Purdue University, 560 Oval Drive, West Lafayette, IN, USA

[2] Purdue Institute for Drug Discovery, Integrative Data Science Institute, West Lafayette, IN, USA; Purdue Center for Cancer Research, Purdue Institute for Drug Discovery, Purdue Institute for Inflammation, Immunology and Infectious Disease, Purdue Institute for Integrative Neuroscience, West Lafayette, IN, USA

* Corresponding authors

Contents

Table S1. Previously published diagnostic product branching ratios and the proton affinities (PA) of the analytes as calculated using an isodesmic reaction scheme and the M06-2x functional using the 6-311++g(d,p) basis set and the given reference analyte (see **Calculation of proton affinities** in the main text for details). Known proton affinity values are supplied where known. The proton affinity calculation is performed for all possible protonation sites and sites with lower proton affinities than the maximum for the analyte are marked as "incorrect protonation". 4

Figure S1. Decision tree for the 40% cutoff model. This model shares some similarities to the 70% cutoff model presented in the main text in that it uses the presence of a sulfoxide group and a N-oxide group as the primary features for the prediction of whether a compound forms a diagnostic addition product (DP) over proton transfer or no reaction (PT). 9

Table S2. This table mirrors **Table 1** in the main text but gives additional details for the reactions of protonated analytes with MOP. The protonation site was determined by calculations of the proton affinities of all basic sites and the formation of a diagnostic product was determined based on data shown in **Figures 2-14.** The proton affinities were calculated using isodesmic reactions and the M06-2x functional using the 6-311++g(d,p) basis set and the given reference analyte (see **Calculation of proton affinities** in the main text for details). 10

Table S3. Additional diagnostic product branching ratio cutoffs and fingerprint radii for the decision tree model. Here, radius refers to the radius parameter of the Morgan algorithm. The compounds are numbered in the same manner as in **Table S2**. 13

Table S4. Regularized logistic regression results for various cutoffs and fingerprint radii. Here, radius refers to the radius parameter of the Morgan algorithm. The compounds are numbered in the same manner as in **Table S2**. 15

Table S5. Generalized Linear Model (GLM) results for various cutoffs and fingerprint radii. Here, radius refers to the radius parameter of the Morgan algorithm. The compounds are numbered in the same manner as in **Table S2**. 17

Table S6. Partial Least Squares (PLS) results for various cutoffs and fingerprint radii. Here, radius refers to the radius parameter of the Morgan algorithm. The compounds are numbered in the same manner as in **Table S2**. 19

Table S7. K-Nearest Neighbor (KNN) results for various cutoffs and fingerprint radii. Here, radius refers to the radius parameter of the Morgan algorithm. The compounds are numbered the same manner as in **Table S2**. 21

Figure S2. MS/MS spectrum measured after 500 ms reaction of protonated diisobutyl sulfoxide with MOP, indicating the formation of a diagnostic addition product (M+H+MOP). 23

Figure S3.MS/MS spectrum measured after 3,000 ms reaction of protonated sulfonyl dimidazole with MOP, indicating the formation of a diagnostic addition product. 23

Figure S4. MS/MS spectrum measured after 10,000 ms reaction of protonated picoline N-oxide with MOP, indicating the formation of a diagnostic addition product. No proton transfer product was observed. 24

Figure S5. MS/MS spectrum measured after 3000 ms reaction of protonated ricobendazole with MOP, indicating the formation of a diagnostic addition product. No proton transfer product was observed. ...24

Figure S6. MS/MS spectrum measured after 10,000 ms reaction of protonated 8-nitroquinolone with MOP, indicating that no diagnostic addition product was formed. No proton transfer product was formed, either. 25

Figure S7. MS/MS spectrum measured after 3,000 ms reaction of protonated benzene sulfonic acid with MOP, indicating that a diagnostic addition product was not formed. Instead, a proton transfer product was observed (MOP+H). 25

Figure S8. MS/MS spectrum measured after 10,000 ms reaction of protonated albendazole with MOP, indicating that a diagnostic addition product was not formed. No proton transfer product was observed, either.. 26

Figure S9. MS/MS spectrum measured after 3,000 ms reaction of protonated 4-nitroquinoline N-oxide with MOP. Although evidence of a diagnostic addition product is seen, the presence of a major proton transfer product indicates that this reaction is not suitable for diagnostic applications 26

Figure S10. MS/MS spectrum measured after 3,000 ms reaction of protonated 3-methylbenzophenone with MOP, indicating that a diagnostic addition product was not formed. Instead, a proton transfer product was observed.. 27

Figure S11. MS/MS spectrum measured after 3,000 ms reaction of protonated 4-nitropyridine N-oxide with MOP, indicating that a diagnostic addition product was not formed. Instead, a proton transfer product was observed. 27

Figure S12. MS/MS spectrum measured after 10,000 ms reaction of protonated 3,5-diiodo-4-pyridine-1-acetic acid with MOP, indicating that a diagnostic addition product was not formed. No proton transfer product was observed, either. 28

Figure S13. MS/MS spectrum measured after 500 ms reaction of protonated 2-methoxybenzoic acid with MOP, indicating that a diagnostic addition product was not formed. Instead, a proton transfer product was observed. 28

Figure S14. MS/MS spectrum measured after 51000 ms reaction of protonated 4-methylpyridine N-oxide with MOP, indicating the formation of a diagnostic addition product. No proton transfer product was observed. 29

Table S1. Previously published diagnostic product branching ratios (determined as the percentage of the addition product abundance divided by all product abundances) and the proton affinities (PA) of the analytes as calculated using an isodesmic reaction scheme (the reference compound is also shown) and the M06-2x functional using the 6-311++G(d,p) basis set and the given reference analyte (see **calculation of proton affinities** in the main text for details). Known proton affinity values are supplied where known. The proton affinity calculation is performed for all possible protonation sites and sites with lower proton affinities than the maximum for the analyte are marked as “incorrect protonation”.

Protonated analyte #	Training Set Reaction	Diagnostic Product Branching Ratio [a]	Calculated Proton Affinity (kcal/mol) & Reference	Experimental Proton Affinity (kcal/mol) [b]
1a	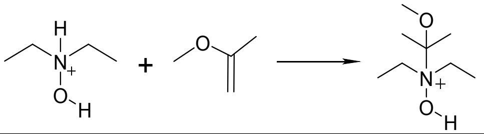	85%	220.2 [c] Ammonia	
1b	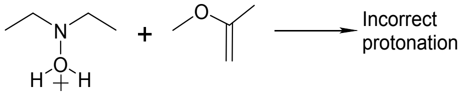		188.2 Methanol	
2a	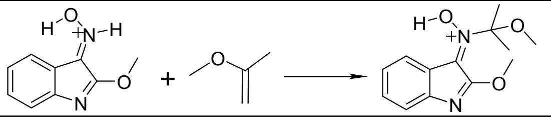	11%	206.7 Ammonia	
2b	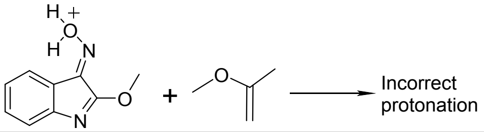		177.0 Methanol	
3	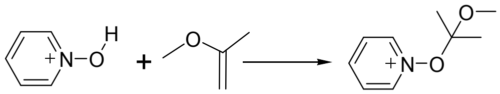	99%	221.4 [c] Methanol	
4	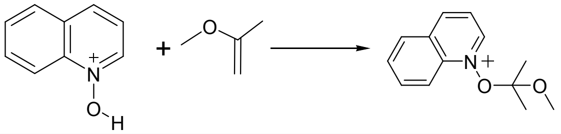	86%	226.2 [c] Methanol	
5	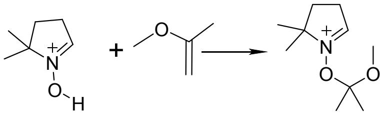	66% [c]	224.7 [c] Methanol	
6	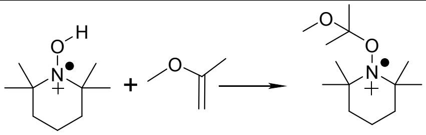	50% [c]	212.6 Methanol	
7a	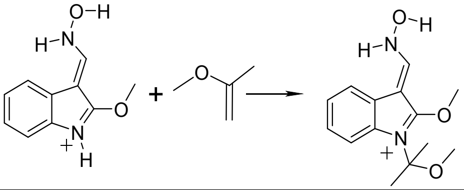	84%	236.0 [d] Ammonia	

7b	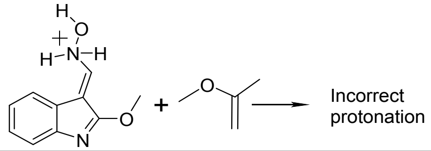			194.0 Ammonia	
8a	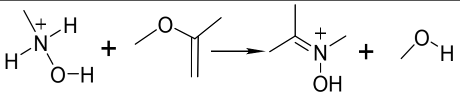		3%	204.2 Ammonia	
8b	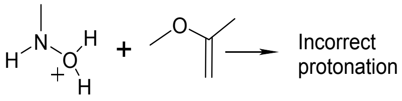			175.8 Methanol	
9a	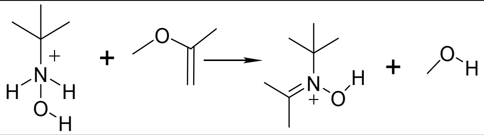		27%	213.9 Ammonia	
9b	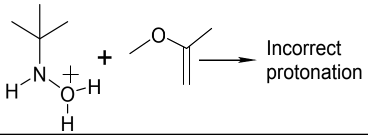			182.1 Methanol	
10a	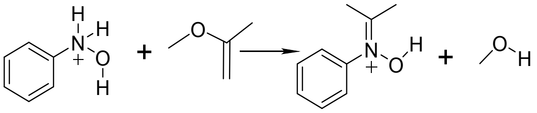		13%	205.8 Ammonia	
10b	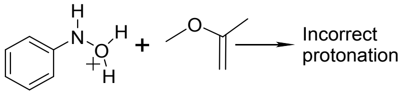			204.9 Methanol	
11a	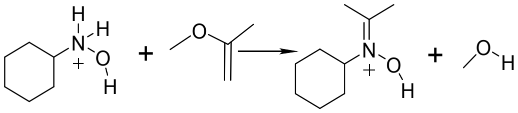		25%	214.3 Ammonia	
11b	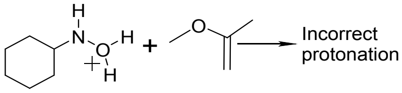			184.7 Ammonia	
12a	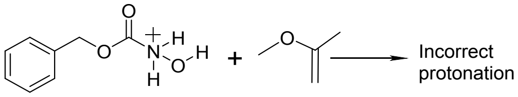 same here			189.2 Ammonia	
12b	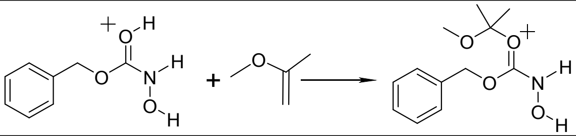		23%	205.2 Methanol	
13a	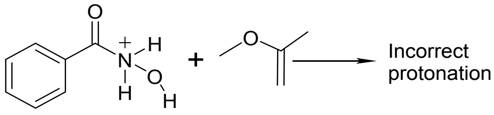			195.8 Ammonia	
13b	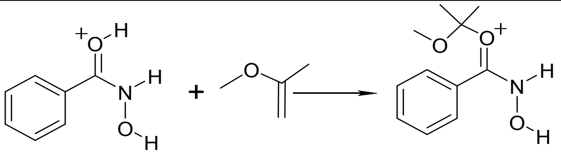		12%	209.7 Methanol	

14	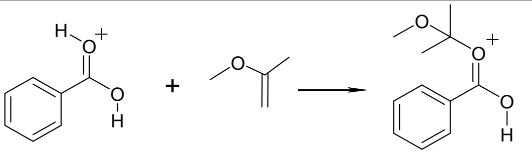	0%	196.2<br>Methanol	196.2
15	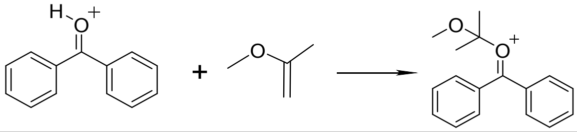	2%	211.3<br>Methanol	210.9
16	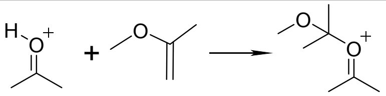	0%	194.4<br>Methanol	194.0
17	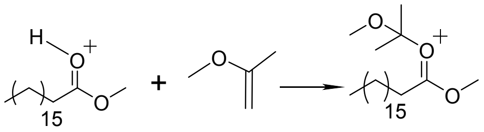	0%	207.9<br>Methanol	
18	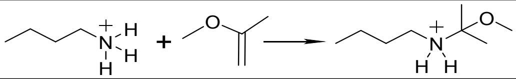	2%	220.0[c]<br>Ammonia	220.2
19	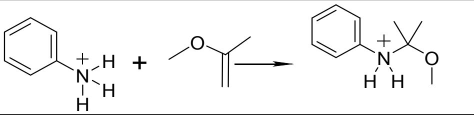	0%	209.6<br>Ammonia	210.9
20	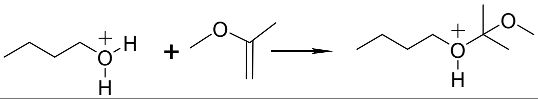	0%	188.0<br>Methanol	188.6
21a	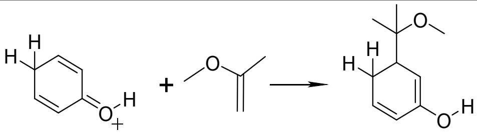	2%	194.0<br>Benzene	195.3
21b	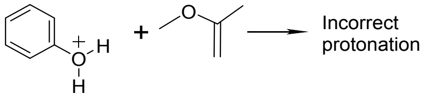		178.0<br>Methanol	
22	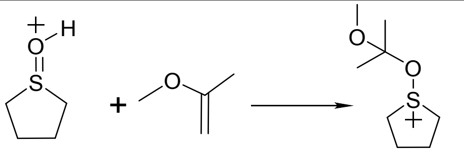	37%	220.9[d]<br>Methanol	
23	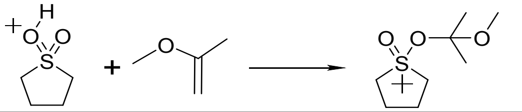	0%	199.7<br>Methanol	
24	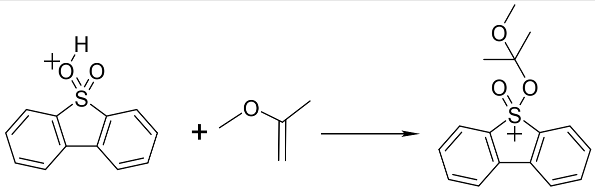	0%	205.2<br>Methanol	
25	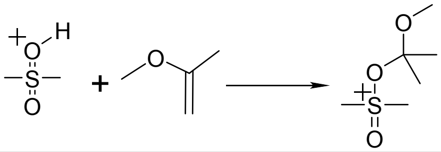	0%	195.5<br>Methanol	

26	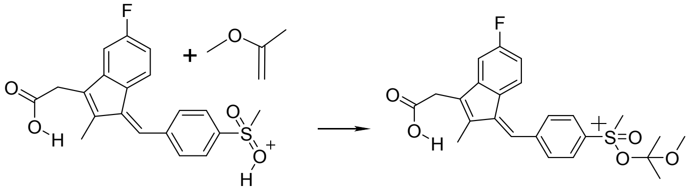	15%	200.8 Methanol	
27	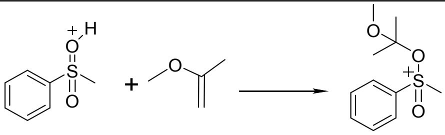	1%	202.0 Methanol	
28	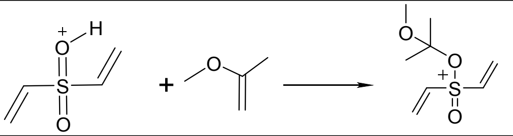	1%	196.9 Methanol	
29	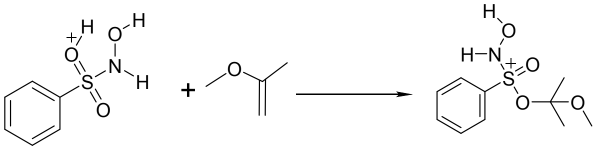	2%	195.2 Methanol	
30	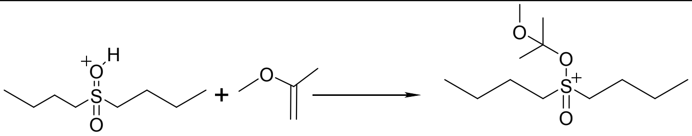	1%	200.8 Methanol	
31	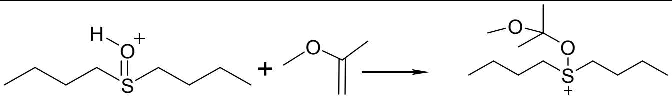	99%	223.9 [c] Methanol	
32	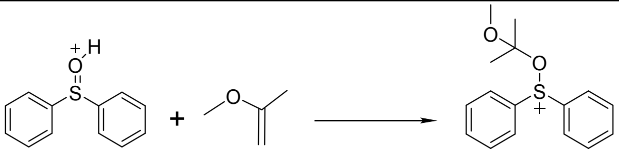	99%	223.2 [c] Methanol	
33	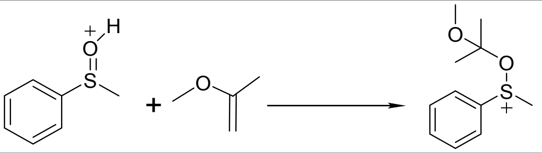	55%[d]	221.1 [c] Methanol	
34	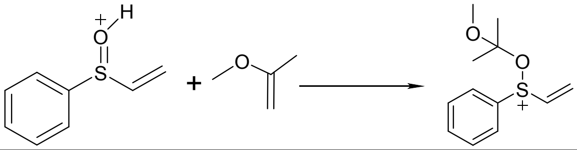	50%[d]	222.9 [c] Methanol	
35	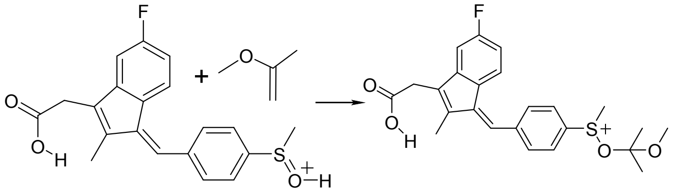	97%	222.0 [c] Methanol	

36	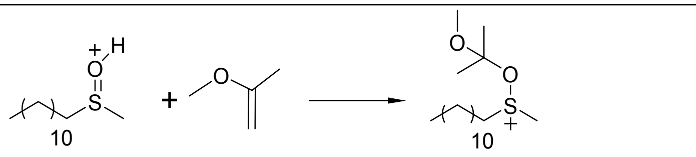		98%	222.8[c] Methanol	

[a] Taken from the following:

(1) Sheng, H.; Williams, P. E.; Tang, W.; Zhang, M.; Kenttämaa, H. I. Identification of the Sulfoxide Functionality in Protonated Analytes via Ion/Molecule Reactions in Linear Quadrupole Ion Trap Mass Spectrometry. *Analyst* **2014**, *139*, 4296–4302.

(2) Sheng, H.; Tang, W.; Yerabolu, R.; Kong, J. Y.; Williams, P. E.; Zhang, M.; Kenttämaa, H. I. Mass Spectrometric Identification of the N-Monosubstituted N-Hydroxylamino Functionality in Protonated Analytes via Ion/Molecule Reactions in Tandem Mass Spectrometry. *Rapid Commun. Mass Spectrom.* **2015**, *29*, 730–734.

(3) Habicht, S. C.; Vinueza, N. R.; Archibold, E. F.; Duan, P.; Kenttämaa, H. I. Identification of the Carboxylic Acid Functionality by Using Electrospray Ionization and Ion-Molecule Reactions in a Modified Linear Quadrupole Ion Trap Mass Spectrometer. *Anal. Chem.* **2008**, *80*, 3416–3421.

(4) Campbell, K. M.; Watkins, M. A.; Li, S.; Fiddler, M. N.; Winger, B.; Kenttämaa, H. I. Functional Group Selective Ion/Molecule Reactions: Mass Spectrometric Identification of the Amido Functionality in Protonated Monofunctional Compounds. *J. Org. Chem.* **2007**, *72*, 3159–3165.

[b] Taken from

Hunter, E. P. L.; Lias, S. G. Evaluated Gas Phase Basicities and Proton Affinities of Molecules: An Update. *J. Phys. Chem. Ref. Data* **1998**, *27*, 413–656.

[c] PA is greater than that of MOP (214.42 kcal/mol as calculated using DFT and an isodesmic reaction scheme using 2-methyl propylene as a reference analyte). Note that the experimental value of MOP is 213.9 kcal/mol (taken from Hunter et al [b]). See **Calculation of proton affinities** in the main text for details.

[d] Reactivity change from the 67th to 78th quantile. See **Cutoff Assignments for Machine Learning Model** and **Figure 3** in the main text for details.

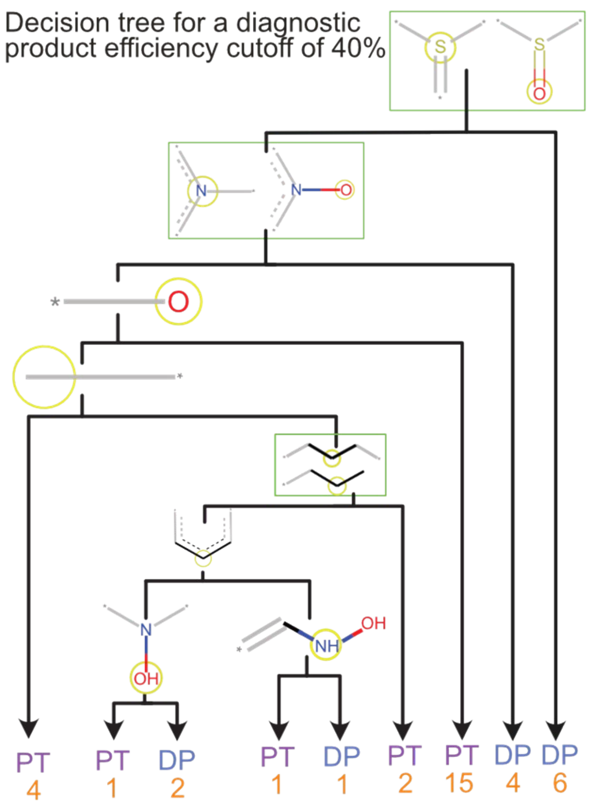

Figure S1. Decision tree for the 40% cutoff model. This model shares some similarities to the 70% cutoff model presented in the main text in that it uses the presence of a sulfoxide group and a N-oxide group as the primary features for the prediction of whether a compound forms a diagnostic addition product (DP) over proton transfer or no reaction (PT).

Table S2. This table mirrors **Table 1** in the main text but gives additional details for the reactions of protonated analytes with MOP. The protonation site was determined by calculations of the proton affinities of all basic sites and the formation of a diagnostic product was determined based on data shown in **Figures S2 – S14.** The proton affinities were calculated using the M06-2x functional with the 6-311++g(d,p) basis set. Isodesmic reactions were used and each reference analyte is shown below (see **calculation of proton affinities** in the main text for details).

Test Set Reaction #	Diagnostic **Reactions**	Diagnostic reaction	Proton affinity kcal/mol	Reference
1a	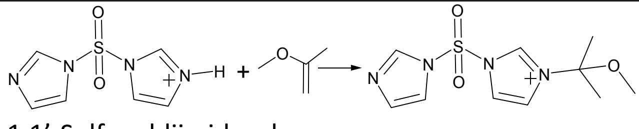 1,1'-Sulfonyldiimidazole	Yes	214.43 [a]	Ammonia
1b	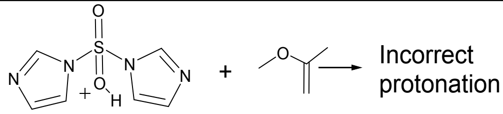 1,1'-Sulfonyldiimidazole		170.12	Methanol
2a	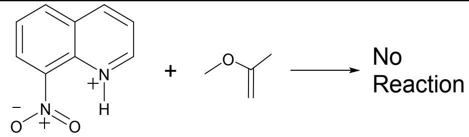 8-Nitroquinoline	No	225.23 [a]	Ammonia
2b	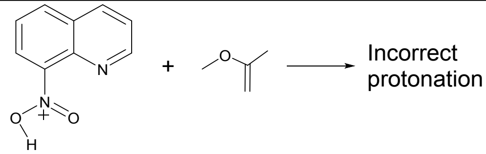 8-Nitroquinoline		199.46	Methanol
3a	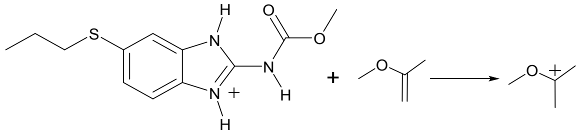 Albendazole	No	229.51 [a]	Ammonia
3b	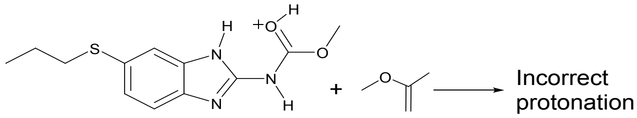 Albendazole		190.26	Methanol
4	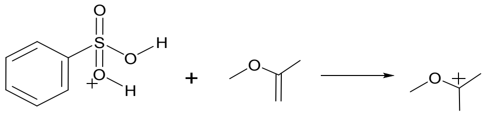 Benzene sulfonic acid	No	188.57	Methanol
5a	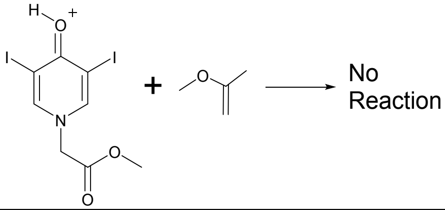	No	222.71 [a]	Methanol

	3,5-Diiodo-4-pyridone-1-acetic acid			
5b	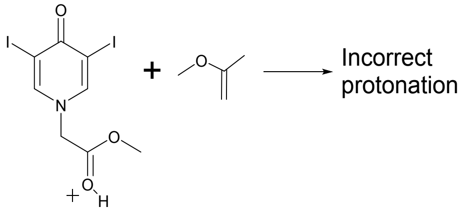<br>3,5-Diiodo-4-pyridone-1-acetic acid		165.34	Methanol
6	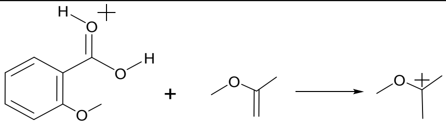<br>2-Methoxybenzoic acid	No	195.01	Methanol
7	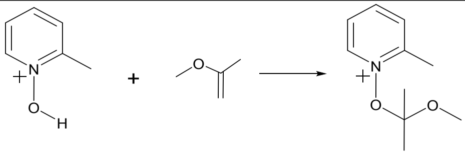<br>2-Methylpyridine N-oxide	Yes	224.15 [a]	Methanol
8	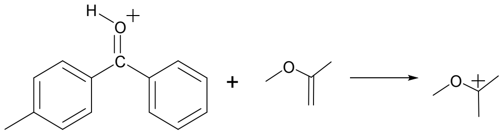<br>3-Methylbenzophenone	No	214.36	Methanol
9a	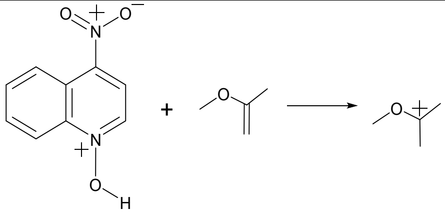<br>4-Nitroquinoline N-oxide	No	213.07	Methanol
9b	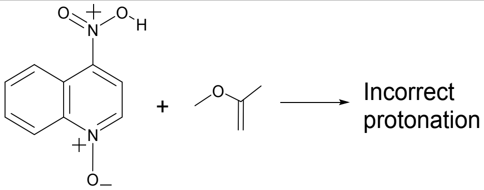<br>4-Nitroquinoline N-oxide		186.45	Methanol
10	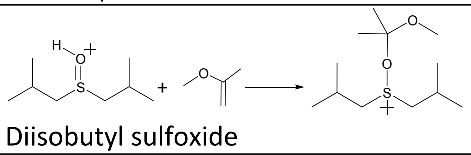<br>Diisobutyl sulfoxide	Yes	228.46[a]	Methanol
11a	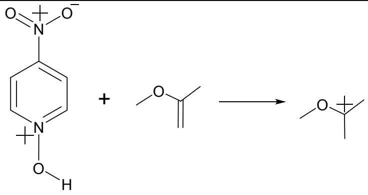<br>4-Nitropyridine N-oxide	No	205.64	Methanol

11b	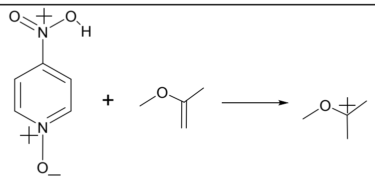<br>4-Nitropyridine N-oxide		179.97	Methanol
12	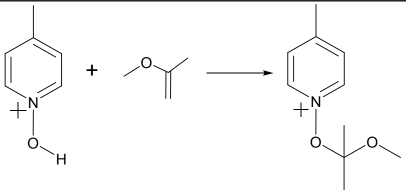<br>4-Methylpyridine N-oxide	Yes	226.38 [a]	Methanol
13a	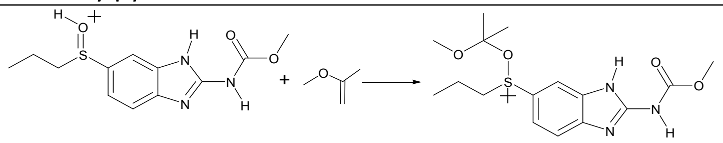<br>Ricobendazole	Yes	232.58 [a]	Methanol
13b	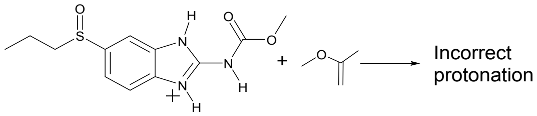<br>Ricobendazole		223.44 [a]	Ammonia

[a] PA is greater than that of MOP (214.42 kcal/mol as calculated using DFT and an isodesmic reaction scheme using 2-methyl propylene as a reference analyte). Note that the experimental value of MOP is 213.9 kcal/mol (see Hunter et al reference below). See **Calculation of proton affinities** in the main text for details.

Hunter, E. P. L.; Lias, S. G. Evaluated Gas Phase Basicities and Proton Affinities of Molecules: An Update. *J. Phys. Chem. Ref. Data* **1998**, *27*, 413–656.

Table S3. Additional diagnostic product branching ratio cutoffs and fingerprint radii for the decision tree model. Here, radius refers to the radius parameter of the Morgan algorithm. The compounds are numbered in the same manner as in **Table S2**.

Compound	Radius	0.1	0.2	0.3	0.4	0.5	0.6	0.7	0.9
01	0	1.00	1.00	1.00	1.00	1.00	1.00	1.00	0.50
02		0.00	0.00	0.50	0.00	0.00	0.00	0.00	0.00
03		1.00	1.00	0.50	0.00	0.00	0.00	0.00	0.00
04		0.00	0.00	0.00	0.00	0.00	0.00	0.00	0.00
05		1.00	1.00	1.00	1.00	1.00	1.00	1.00	0.50
06		1.00	0.00	0.00	0.00	0.00	0.00	0.00	0.00
07		1.00	1.00	1.00	1.00	0.84	0.84	0.66	0.33
08		1.00	0.00	0.00	0.00	0.00	0.00	0.00	0.00
09		1.00	1.00	1.00	1.00	1.00	1.00	1.00	0.50
10		1.00	1.00	1.00	1.00	1.00	1.00	1.00	1.00
11		1.00	1.00	1.00	1.00	1.00	1.00	1.00	0.50
12		1.00	1.00	1.00	1.00	0.84	0.84	0.66	0.33
13		1.00	1.00	1.00	1.00	1.00	1.00	1.00	1.00
kappa		0.20	0.43	0.56	0.56	0.56	0.56	0.56	0.45
01	1	0.50	0.51	0.54	0.50	0.47	1.00	1.00	0.50
02		0.00	0.00	0.08	0.00	0.00	0.00	0.00	0.00
03		0.11	0.00	0.08	0.00	0.00	0.33	0.00	0.00
04		0.00	0.00	0.00	0.00	0.00	0.00	0.00	0.00
05		0.52	0.59	0.58	0.50	0.44	0.04	0.00	0.00
06		0.00	0.00	0.00	0.00	0.00	0.33	0.00	0.00
07		1.00	1.00	1.00	1.00	0.94	1.00	1.00	0.50
08		0.00	0.00	0.00	0.00	0.00	0.33	0.00	0.00
09		1.00	1.00	1.00	1.00	1.00	1.00	1.00	0.50
10		1.00	1.00	1.00	1.00	1.00	0.61	1.00	1.00
11		1.00	1.00	1.00	1.00	0.94	1.00	1.00	0.50
12		1.00	1.00	1.00	1.00	0.88	1.00	1.00	0.50
13		1.00	1.00	1.00	1.00	1.00	1.00	1.00	1.00
kappa		0.40	0.56	0.56	0.56	0.53	0.70	**0.70**	0.45
01	2	0.50	0.51	0.50	0.50	0.47	1.00	1.00	0.25
02		0.00	0.00	0.67	0.00	0.00	0.00	0.00	0.00
03		0.11	0.00	0.00	0.00	0.00	0.35	0.00	0.00
04		0.00	0.00	0.00	0.00	0.00	0.00	0.00	0.00
05		0.52	0.59	0.50	0.50	0.44	0.04	0.00	0.00
06		0.00	0.00	0.00	0.00	0.00	0.33	0.00	0.00
07		1.00	1.00	1.00	1.00	0.94	1.00	1.00	0.25
08		0.00	0.00	0.00	0.00	0.00	0.33	0.00	0.00
09		1.00	1.00	1.00	1.00	1.00	1.00	1.00	0.25
10		1.00	1.00	1.00	1.00	1.00	0.60	1.00	1.00
11		1.00	1.00	1.00	1.00	0.94	1.00	1.00	0.25

12		1.00	1.00	1.00	1.00	0.88	1.00	1.00	0.25
13		1.00	1.00	1.00	1.00	1.00	1.00	1.00	1.00
kappa		0.40	0.56	0.43	0.56	0.53	0.70	0.70	0.45
01	3	0.50	0.49	0.49	0.50	1.00	1.00	1.00	0.25
02		0.49	0.50	1.00	0.50	0.42	0.39	0.38	0.40
03		0.51	0.00	0.28	0.48	0.67	0.68	0.33	0.13
04		0.00	0.00	0.00	0.00	0.00	0.00	0.00	0.00
05		1.00	0.67	0.49	0.50	0.00	0.00	0.00	0.13
06		1.00	0.00	0.00	0.95	1.00	1.00	0.67	0.00
07		1.00	1.00	1.00	1.00	1.00	1.00	1.00	0.25
08		0.00	0.00	0.00	0.95	1.00	1.00	0.67	0.00
09		1.00	1.00	1.00	1.00	1.00	1.00	1.00	0.25
10		1.00	1.00	1.00	1.00	1.00	1.00	1.00	1.00
11		1.00	1.00	1.00	1.00	1.00	1.00	1.00	0.25
12		1.00	1.00	1.00	1.00	1.00	1.00	1.00	0.25
13		1.00	1.00	1.00	1.00	1.00	1.00	1.00	1.00
kappa		0.32	0.40	0.40	0.27	0.32	0.32	0.43	0.45

Table S4. Regularized logistic regression results for various cutoffs and fingerprint radii. Here, radius refers to the radius parameter of the Morgan algorithm. The compounds are numbered in the same manner as in **Table S2**.

Compound	Radius	0.1	0.2	0.3	0.4	0.5	0.6	0.7	0.9
01	0	0.63	0.46	0.55	0.69	0.77	0.33	0.27	0.21
02		0.82	0.38	0.38	0.22	0.67	0.33	0.27	0.11
03		0.89	0.36	0.21	0.19	0.23	0.28	0.27	0.03
04		0.13	0.14	0.10	0.15	0.10	0.28	0.27	0.03
05		0.78	0.67	0.56	0.71	0.65	0.28	0.27	0.18
06		0.36	0.36	0.21	0.19	0.11	0.28	0.27	0.03
07		0.79	0.69	0.75	0.75	0.65	0.33	0.27	0.11
08		0.36	0.36	0.21	0.19	0.11	0.28	0.27	0.03
09		0.90	0.69	0.75	0.75	0.93	0.33	0.27	0.17
10		0.81	0.86	0.86	0.84	0.80	0.34	0.33	0.70
11		0.90	0.69	0.75	0.75	0.93	0.33	0.27	0.17
12		0.79	0.69	0.75	0.75	0.65	0.33	0.27	0.11
13		0.98	0.80	0.71	0.81	0.90	0.29	0.33	0.14
kappa		0.32	0.40	0.56	0.56	0.43	0.00	0.00	0.24
01	1	0.56	0.48	0.48	0.45	0.39	0.33	0.27	0.20
02		0.65	0.48	0.41	0.22	0.16	0.33	0.27	0.08
03		0.65	0.37	0.19	0.18	0.08	0.28	0.27	0.02
04		0.13	0.10	0.08	0.16	0.08	0.28	0.27	0.02
05		0.61	0.41	0.48	0.46	0.24	0.28	0.27	0.18
06		0.32	0.17	0.19	0.18	0.08	0.28	0.27	0.04
07		0.83	0.67	0.72	0.75	0.74	0.33	0.27	0.12
08		0.29	0.17	0.19	0.18	0.08	0.28	0.27	0.03
09		0.86	0.74	0.72	0.75	0.75	0.33	0.27	0.15
10		0.84	0.81	0.87	0.84	0.77	0.34	0.33	0.60
11		0.85	0.72	0.72	0.75	0.75	0.33	0.27	0.31
12		0.84	0.68	0.72	0.75	0.74	0.33	0.27	0.24
13		0.95	0.79	0.65	0.80	0.60	0.29	0.33	0.18
kappa		0.32	0.53	0.53	0.53	0.53	0.00	0.00	0.24
01	2	0.53	0.48	0.25	0.28	0.26	0.33	0.27	0.18
02		0.63	0.49	0.39	0.22	0.27	0.33	0.27	0.18
03		0.64	0.36	0.20	0.18	0.21	0.28	0.27	0.17
04		0.12	0.10	0.09	0.16	0.14	0.28	0.27	0.15
05		0.56	0.42	0.24	0.29	0.29	0.28	0.27	0.17
06		0.26	0.16	0.20	0.18	0.20	0.28	0.27	0.17
07		0.81	0.67	0.75	0.75	0.52	0.33	0.27	0.17
08		0.19	0.13	0.20	0.18	0.20	0.28	0.27	0.15
09		0.87	0.76	0.75	0.75	0.52	0.33	0.27	0.17
10		0.81	0.79	0.86	0.84	0.66	0.34	0.33	0.35
11		0.85	0.74	0.75	0.75	0.52	0.33	0.27	0.18

12		0.82	0.69	0.75	0.75	0.52	0.33	0.27	0.18
13		0.94	0.78	0.71	0.80	0.59	0.29	0.33	0.32
kappa		0.32	0.53	0.53	0.53	0.53	0.00	0.00	0.00
01	3	0.44	0.42	0.46	0.51	0.34	0.47	0.51	0.23
02		0.85	0.59	0.81	0.63	0.68	0.58	0.50	0.48
03		0.33	0.30	0.29	0.27	0.31	0.32	0.26	0.17
04		0.09	0.12	0.08	0.13	0.10	0.01	0.02	0.07
05		0.78	0.68	0.61	0.65	0.39	0.59	0.26	0.18
06		0.63	0.38	0.35	0.29	0.33	0.36	0.26	0.18
07		0.87	0.68	0.75	0.75	0.59	0.81	0.75	0.09
08		0.38	0.31	0.21	0.20	0.17	0.06	0.06	0.07
09		0.70	0.67	0.68	0.63	0.59	0.81	0.52	0.09
10		0.98	0.91	0.89	0.87	0.75	0.77	0.68	0.62
11		0.81	0.68	0.75	0.75	0.59	0.82	0.79	0.23
12		0.87	0.68	0.75	0.75	0.59	0.81	0.75	0.23
13		0.93	0.80	0.80	0.85	0.73	0.73	0.68	0.60
kappa		0.15	0.27	0.27	0.43	0.40	0.27	0.70	0.45

Table S5. Generalized Linear Model (GLM) results for various cutoffs and fingerprint radii. Here, radius refers to the radius parameter of the Morgan algorithm. The compounds are numbered in the same manner as in **Table S2**.

Compound	Radius	0.1	0.2	0.3	0.4	0.5	0.6	0.7	0.9
01	0	0.00	1.00	1.00	1.00	1.00	1.00	1.00	1.00
02		1.00	1.00	1.00	1.00	1.00	1.00	1.00	0.00
03		0.00	0.98	1.00	1.00	1.00	0.00	0.00	0.00
04		0.00	0.00	0.00	0.00	0.00	0.00	0.00	0.00
05		0.00	0.20	1.00	1.00	1.00	1.00	1.00	0.00
06		0.00	0.00	0.00	0.00	0.00	0.00	0.00	0.00
07		0.00	1.00	1.00	1.00	1.00	0.00	0.00	0.00
08		0.00	0.00	0.00	0.00	0.00	0.00	0.00	0.00
09		1.00	1.00	1.00	1.00	1.00	1.00	1.00	0.00
10		1.00	1.00	1.00	1.00	1.00	1.00	1.00	1.00
11		1.00	1.00	1.00	1.00	1.00	1.00	1.00	0.00
12		0.00	1.00	1.00	1.00	1.00	0.00	0.00	0.00
13		0.00	1.00	1.00	1.00	1.00	1.00	1.00	0.00
kappa		-0.18	0.43	0.32	0.32	0.32	0.09	0.09	0.45
01	1	0.00	0.00	0.00	0.00	0.00	0.00	0.00	0.00
02		1.00	1.00	1.00	1.00	0.00	0.00	0.00	0.00
03		0.00	0.00	0.00	0.00	0.00	0.00	0.00	0.00
04		0.00	0.00	0.00	0.00	0.00	0.00	0.00	0.00
05		1.00	1.00	1.00	1.00	1.00	1.00	1.00	1.00
06		0.00	0.00	0.00	0.00	0.00	0.00	0.00	0.00
07		1.00	1.00	1.00	1.00	1.00	1.00	1.00	1.00
08		0.00	0.00	0.00	0.00	0.00	0.00	0.00	0.00
09		1.00	1.00	1.00	1.00	1.00	1.00	1.00	0.00
10		1.00	1.00	1.00	1.00	1.00	1.00	1.00	1.00
11		1.00	1.00	1.00	1.00	1.00	1.00	1.00	1.00
12		1.00	1.00	1.00	1.00	1.00	1.00	1.00	1.00
13		0.00	0.00	0.00	0.00	0.00	0.00	0.00	0.00
kappa		0.09	0.09	0.09	0.09	0.22	0.22	0.22	0.35
01	2	1.00	1.00	1.00	1.00	0.00	0.00	0.00	0.00
02		1.00	1.00	1.00	1.00	0.00	0.00	0.00	0.00
03		1.00	1.00	1.00	1.00	0.00	0.00	0.00	0.00
04		0.00	0.00	0.00	0.00	0.00	0.00	0.00	0.00
05		0.00	0.00	0.00	0.00	0.00	0.00	0.00	0.00
06		0.00	0.00	0.00	0.00	0.00	0.00	0.00	0.00
07		0.00	0.00	0.00	0.00	0.00	0.00	0.00	0.00
08		0.00	0.00	0.00	0.00	0.00	0.00	0.00	0.00
09		1.00	1.00	1.00	1.00	1.00	1.00	1.00	0.00
10		1.00	1.00	1.00	1.00	1.00	0.00	0.00	0.00
11		1.00	1.00	1.00	1.00	1.00	1.00	1.00	1.00

12		1.00	1.00	1.00	1.00	1.00	1.00	1.00	1.00
13		1.00	1.00	1.00	1.00	1.00	1.00	1.00	1.00
kappa		0.27	0.27	0.27	0.27	0.35	0.16	0.16	0.30
01	3	0.00	0.00	0.00	0.00	0.00	0.00	0.00	0.00
02		1.00	1.00	1.00	1.00	0.00	0.00	0.00	0.00
03		1.00	1.00	1.00	1.00	0.00	0.00	0.00	0.00
04		0.00	0.00	0.00	0.00	0.00	0.00	0.00	0.00
05		0.00	1.00	1.00	1.00	1.00	0.00	0.00	0.00
06		1.00	1.00	1.00	1.00	1.00	0.00	0.00	0.00
07		1.00	1.00	1.00	1.00	1.00	1.00	1.00	1.00
08		0.00	0.00	0.00	0.00	0.00	0.00	0.00	0.00
09		0.00	0.00	0.00	0.00	0.00	0.00	0.00	0.00
10		1.00	1.00	1.00	1.00	1.00	1.00	1.00	1.00
11		1.00	1.00	1.00	1.00	1.00	1.00	1.00	1.00
12		1.00	1.00	1.00	1.00	1.00	1.00	1.00	1.00
13		1.00	1.00	1.00	1.00	0.00	0.00	0.00	0.00
kappa		0.27	0.15	0.15	0.15	0.22	0.49	0.49	0.49

Table S6. Partial Least Squares (PLS) results for various cutoffs and fingerprint radii. Here, radius refers to the radius parameter of the Morgan algorithm. The compounds are numbered in the same manner as in **Table S2**.

Compound	Radius	0.1	0.2	0.3	0.4	0.5	0.6	0.7	0.9
01	0	0.60	0.42	0.47	0.51	0.59	0.65	0.38	0.38
02		0.68	0.53	0.60	0.51	0.60	0.59	0.43	0.30
03		0.77	0.62	0.49	0.36	0.49	0.50	0.44	0.31
04		0.21	0.14	0.17	0.26	0.14	0.14	0.24	0.19
05		0.66	0.56	0.53	0.48	0.52	0.61	0.46	0.41
06		0.33	0.35	0.40	0.40	0.32	0.26	0.35	0.24
07		0.64	0.63	0.69	0.66	0.66	0.60	0.46	0.32
08		0.33	0.35	0.40	0.40	0.32	0.26	0.35	0.24
09		0.79	0.68	0.70	0.62	0.73	0.73	0.46	0.35
10		0.68	0.74	0.71	0.69	0.71	0.64	0.62	0.68
11		0.79	0.68	0.70	0.62	0.73	0.73	0.46	0.35
12		0.64	0.63	0.69	0.66	0.66	0.60	0.46	0.32
13		0.91	0.85	0.74	0.54	0.74	0.70	0.59	0.58
kappa		0.32	0.15	0.27	0.56	0.43	0.32	0.45	0.45
01	1	0.55	0.50	0.48	0.46	0.45	0.57	0.60	0.33
02		0.58	0.47	0.55	0.48	0.48	0.42	0.48	0.33
03		0.57	0.50	0.38	0.37	0.38	0.38	0.37	0.28
04		0.27	0.18	0.23	0.25	0.22	0.21	0.23	0.23
05		0.49	0.43	0.39	0.40	0.37	0.44	0.43	0.35
06		0.30	0.22	0.32	0.35	0.29	0.23	0.24	0.29
07		0.71	0.64	0.69	0.65	0.69	0.67	0.67	0.41
08		0.30	0.23	0.32	0.34	0.31	0.23	0.22	0.28
09		0.78	0.70	0.72	0.63	0.72	0.77	0.80	0.41
10		0.70	0.74	0.65	0.65	0.61	0.61	0.60	0.54
11		0.75	0.67	0.68	0.61	0.67	0.74	0.75	0.45
12		0.71	0.64	0.68	0.64	0.66	0.67	0.66	0.44
13		0.83	0.81	0.68	0.65	0.66	0.61	0.61	0.54
kappa		0.43	0.40	0.40	0.53	0.53	0.70	0.70	0.45
01	2	0.50	0.45	0.47	0.46	0.42	0.40	0.40	0.31
02		0.54	0.51	0.52	0.50	0.46	0.39	0.45	0.30
03		0.55	0.43	0.34	0.36	0.36	0.39	0.34	0.31
04		0.27	0.22	0.25	0.24	0.23	0.22	0.22	0.26
05		0.50	0.45	0.38	0.37	0.35	0.37	0.34	0.34
06		0.33	0.29	0.31	0.30	0.30	0.29	0.27	0.31
07		0.69	0.65	0.68	0.67	0.65	0.52	0.59	0.33
08		0.30	0.27	0.30	0.30	0.29	0.27	0.25	0.28
09		0.75	0.69	0.71	0.67	0.65	0.50	0.63	0.30
10		0.65	0.67	0.63	0.62	0.56	0.51	0.52	0.43
11		0.70	0.65	0.65	0.63	0.61	0.50	0.58	0.35

12		0.67	0.64	0.64	0.64	0.61	0.52	0.54	0.36
13		0.78	0.72	0.61	0.61	0.60	0.55	0.56	0.44
kappa		0.27	0.40	0.40	0.53	0.53	0.53	0.53	0.00
01	3	0.54	0.48	0.50	0.48	0.45	0.44	0.47	0.34
02		0.60	0.57	0.56	0.56	0.52	0.42	0.51	0.33
03		0.60	0.49	0.45	0.42	0.41	0.43	0.35	0.33
04		0.26	0.22	0.24	0.25	0.24	0.22	0.26	0.26
05		0.58	0.53	0.51	0.44	0.44	0.42	0.44	0.35
06		0.47	0.40	0.42	0.38	0.38	0.35	0.41	0.33
07		0.66	0.63	0.65	0.66	0.63	0.50	0.60	0.34
08		0.33	0.27	0.32	0.31	0.30	0.26	0.32	0.29
09		0.64	0.61	0.59	0.59	0.57	0.46	0.58	0.30
10		0.67	0.69	0.69	0.64	0.59	0.54	0.57	0.45
11		0.65	0.63	0.64	0.61	0.59	0.49	0.61	0.36
12		0.66	0.64	0.64	0.63	0.61	0.53	0.59	0.38
13		0.78	0.72	0.70	0.64	0.62	0.55	0.54	0.43
kappa		0.32	0.27	0.27	0.40	0.40	0.83	0.40	0.00

Table S7. K-Nearest Neighbor (KNN) results for various cutoffs and fingerprint radii. Here, radius refers to the radius parameter of the Morgan algorithm. The compounds are numbered the same manner as in **Table S2**.

Compound	Radius	0.1	0.2	0.3	0.4	0.5	0.6	0.7	0.9
01	0	0.33	0.33	0.33	0.33	0.33	0.33	0.25	0.17
02		0.45	0.36	0.36	0.36	0.36	0.27	0.27	0.18
03		0.56	0.22	0.11	0.11	0.11	0.11	0.11	0.00
04		0.40	0.20	0.20	0.20	0.20	0.20	0.20	0.10
05		0.57	0.43	0.29	0.29	0.29	0.27	0.27	0.18
06		0.29	0.14	0.14	0.14	0.14	0.17	0.17	0.08
07		0.56	0.56	0.56	0.56	0.56	0.44	0.33	0.22
08		0.29	0.14	0.14	0.14	0.14	0.17	0.17	0.08
09		0.45	0.36	0.36	0.36	0.36	0.27	0.27	0.18
10		0.33	0.33	0.33	0.33	0.33	0.22	0.22	0.22
11		0.45	0.36	0.36	0.36	0.36	0.27	0.27	0.18
12		0.56	0.56	0.56	0.56	0.56	0.44	0.33	0.22
13		0.86	0.57	0.43	0.43	0.43	0.36	0.36	0.29
kappa		0.35	0.65	0.45	0.45	0.45	0.00	0.00	0.00
01	1	0.33	0.33	0.29	0.27	0.20	0.20	0.20	0.13
02		0.45	0.36	0.29	0.36	0.36	0.27	0.27	0.18
03		0.33	0.11	0.00	0.00	0.00	0.00	0.00	0.00
04		0.25	0.08	0.13	0.08	0.08	0.08	0.08	0.08
05		0.50	0.30	0.30	0.30	0.30	0.30	0.30	0.20
06		0.40	0.20	0.20	0.20	0.20	0.10	0.10	0.10
07		0.40	0.40	0.50	0.40	0.40	0.30	0.30	0.20
08		0.40	0.20	0.20	0.20	0.20	0.10	0.10	0.10
09		0.45	0.36	0.38	0.36	0.36	0.27	0.27	0.18
10		0.45	0.45	0.45	0.45	0.45	0.36	0.36	0.27
11		0.45	0.36	0.38	0.36	0.36	0.27	0.27	0.18
12		0.33	0.33	0.50	0.33	0.33	0.25	0.25	0.17
13		0.55	0.45	0.57	0.36	0.36	0.27	0.27	0.27
kappa		0.24	0.00	0.24	0.00	0.00	0.00	0.00	0.00
01	2	0.30	0.20	0.20	0.20	0.20	0.20	0.20	0.10
02		0.33	0.14	0.14	0.14	0.33	0.33	0.33	0.22
03		0.11	0.11	0.11	0.11	0.11	0.11	0.11	0.00
04		0.22	0.00	0.00	0.00	0.11	0.11	0.11	0.11
05		0.42	0.20	0.20	0.20	0.25	0.25	0.25	0.17
06		0.33	0.00	0.00	0.00	0.17	0.08	0.08	0.08
07		0.36	0.36	0.36	0.36	0.36	0.27	0.27	0.18
08		0.40	0.00	0.00	0.00	0.20	0.10	0.10	0.10
09		0.38	0.43	0.43	0.43	0.31	0.23	0.23	0.15
10		0.44	0.40	0.40	0.40	0.33	0.33	0.33	0.22
11		0.30	0.33	0.33	0.33	0.30	0.30	0.30	0.20

12		0.33	0.29	0.29	0.29	0.33	0.25	0.25	0.17
13		0.38	0.50	0.50	0.50	0.38	0.31	0.31	0.23
kappa		0.00	0.00	0.00	0.00	0.00	0.00	0.00	0.00
01	3	0.40	0.56	0.44	0.40	0.33	0.33	0.33	0.22
02		0.20	0.40	0.40	0.20	0.40	0.30	0.30	0.30
03		0.40	0.43	0.27	0.40	0.27	0.27	0.27	0.18
04		0.00	0.00	0.10	0.00	0.10	0.10	0.10	0.10
05		0.29	0.29	0.27	0.29	0.27	0.27	0.27	0.18
06		0.17	0.22	0.11	0.17	0.11	0.11	0.11	0.11
07		0.40	0.33	0.33	0.40	0.33	0.22	0.22	0.22
08		0.40	0.22	0.22	0.20	0.22	0.11	0.11	0.11
09		0.40	0.43	0.33	0.40	0.33	0.33	0.33	0.22
10		0.60	0.50	0.33	0.60	0.33	0.33	0.33	0.22
11		0.40	0.38	0.33	0.40	0.25	0.25	0.25	0.17
12		0.43	0.43	0.33	0.29	0.33	0.33	0.33	0.22
13		0.50	0.56	0.44	0.50	0.44	0.33	0.33	0.22
kappa		0.24	0.45	0.00	0.24	0.00	0.00	0.00	0.00

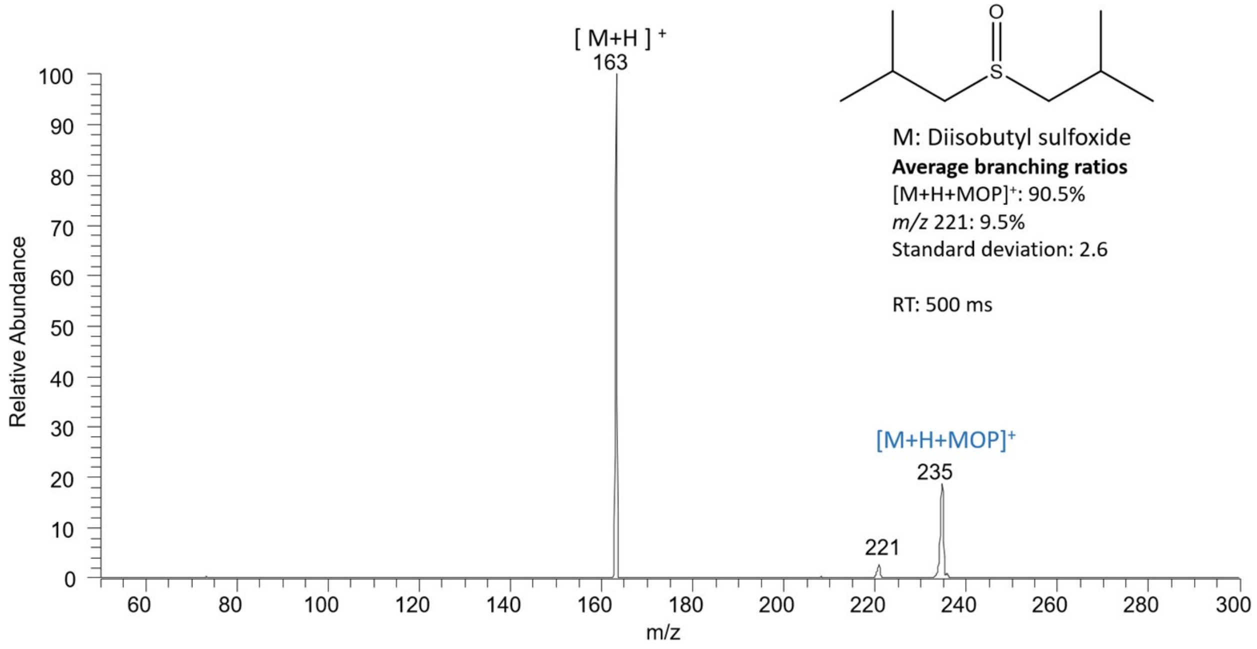

Figure S2. MS/MS spectrum measured after 500 ms reaction of protonated diisobutyl sulfoxide with MOP, indicating the formation of a diagnostic addition product (M+H+MOP).

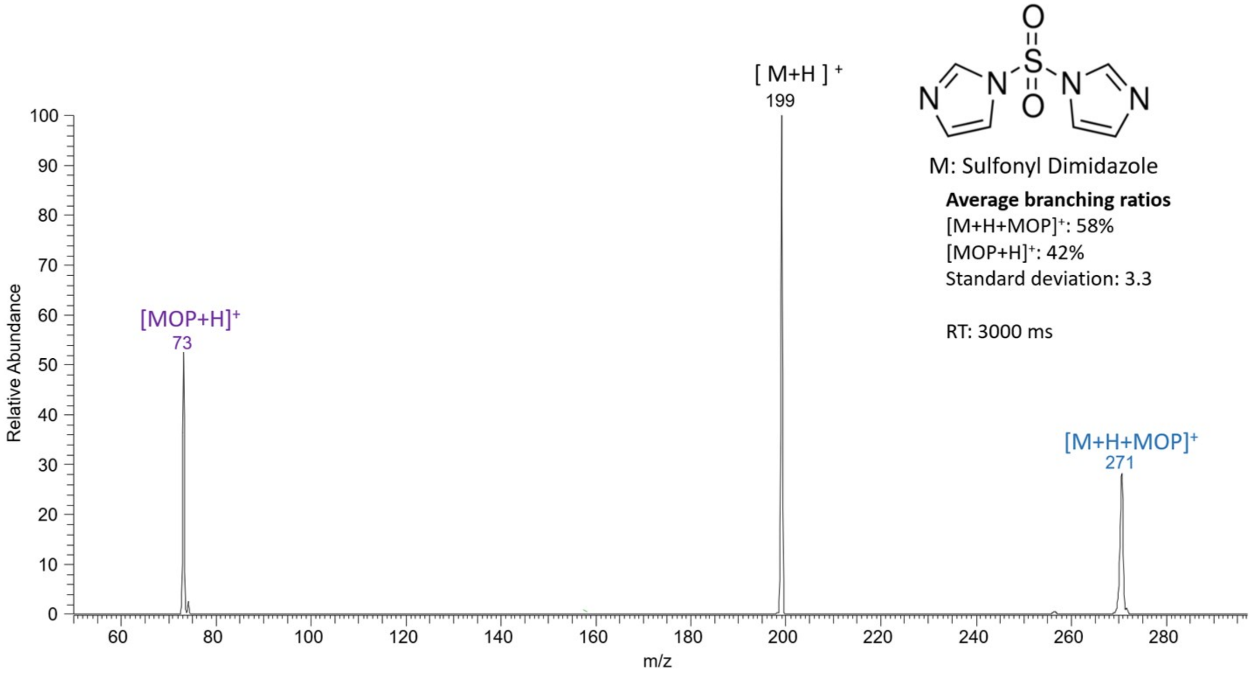

Figure S3.MS/MS spectrum measured after 3,000 ms reaction of protonated sulfonyl dimidazole with MOP, indicating the formation of a diagnostic addition product.

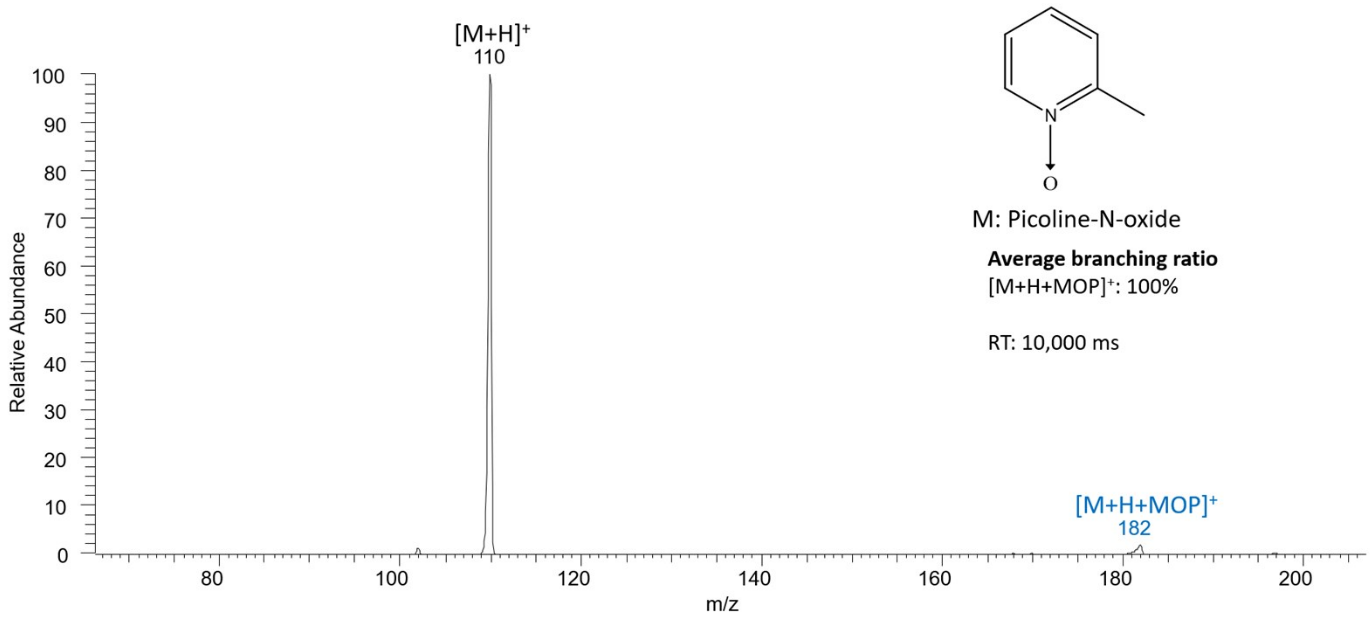

Figure S4. MS/MS spectrum measured after 10,000 ms reaction of protonated picoline N-oxide with MOP, indicating the formation of a diagnostic addition product. No proton transfer product was observed.

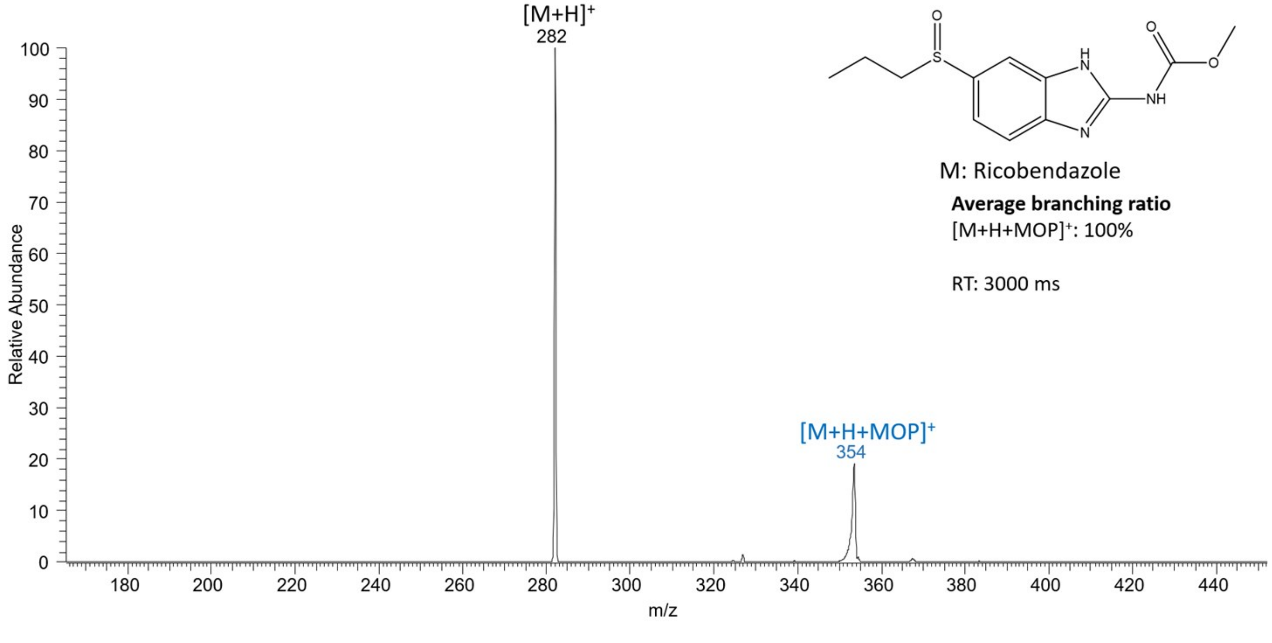

Figure S5. MS/MS spectrum measured after 3000 ms reaction of protonated ricobendazole with MOP, indicating the formation of a diagnostic addition product. No proton transfer product was observed.

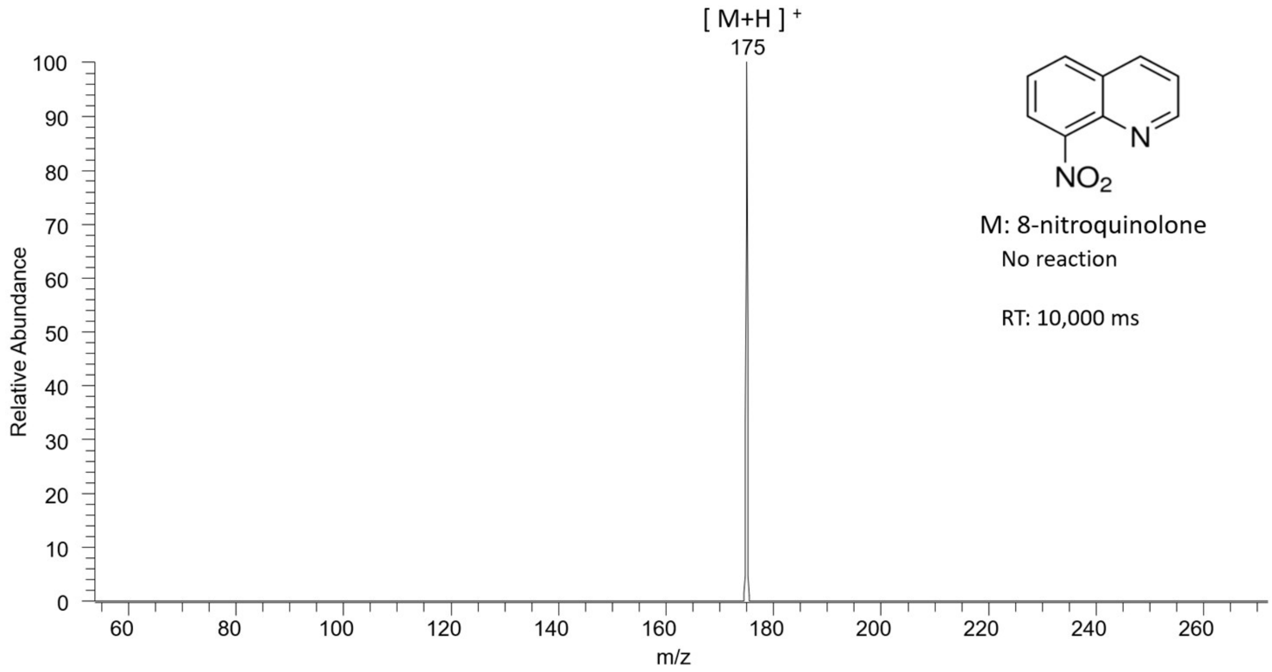

Figure S6. MS/MS spectrum measured after 10,000 ms reaction of protonated 8-nitroquinolone with MOP, indicating that no diagnostic addition product was formed. No proton transfer product was formed, either.

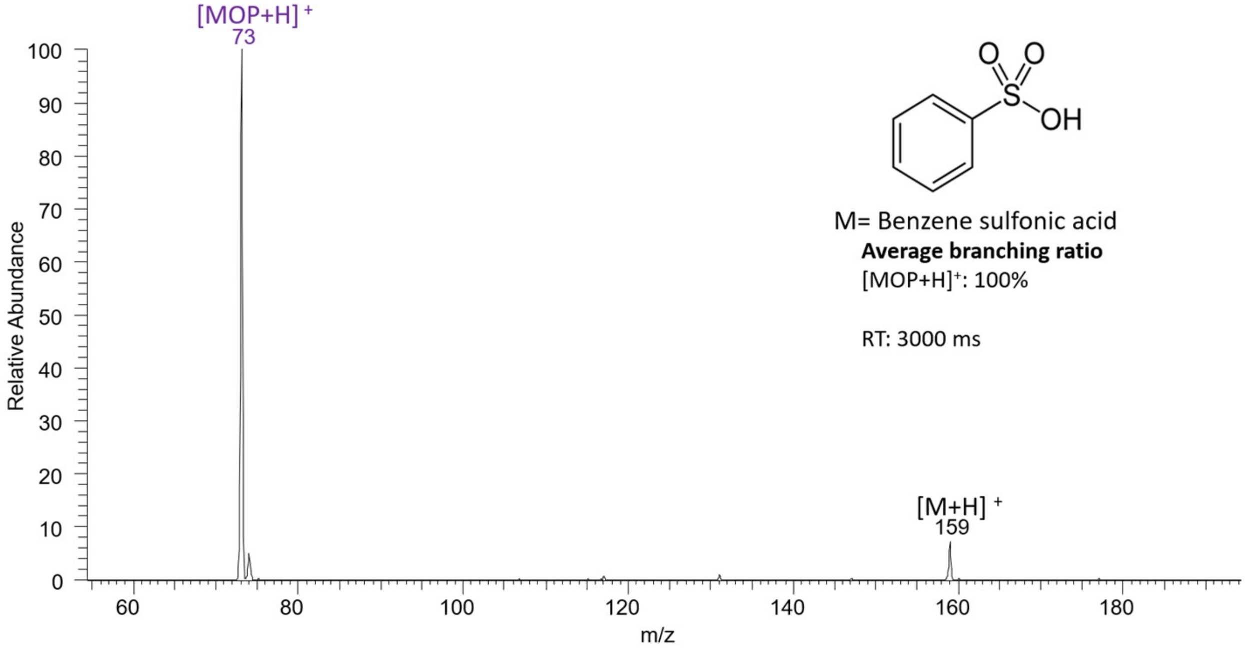

Figure S7. MS/MS spectrum measured after 3,000 ms reaction of protonated benzene sulfonic acid with MOP, indicating that a diagnostic addition product was not formed. Instead, a proton transfer product was observed (MOP+H).

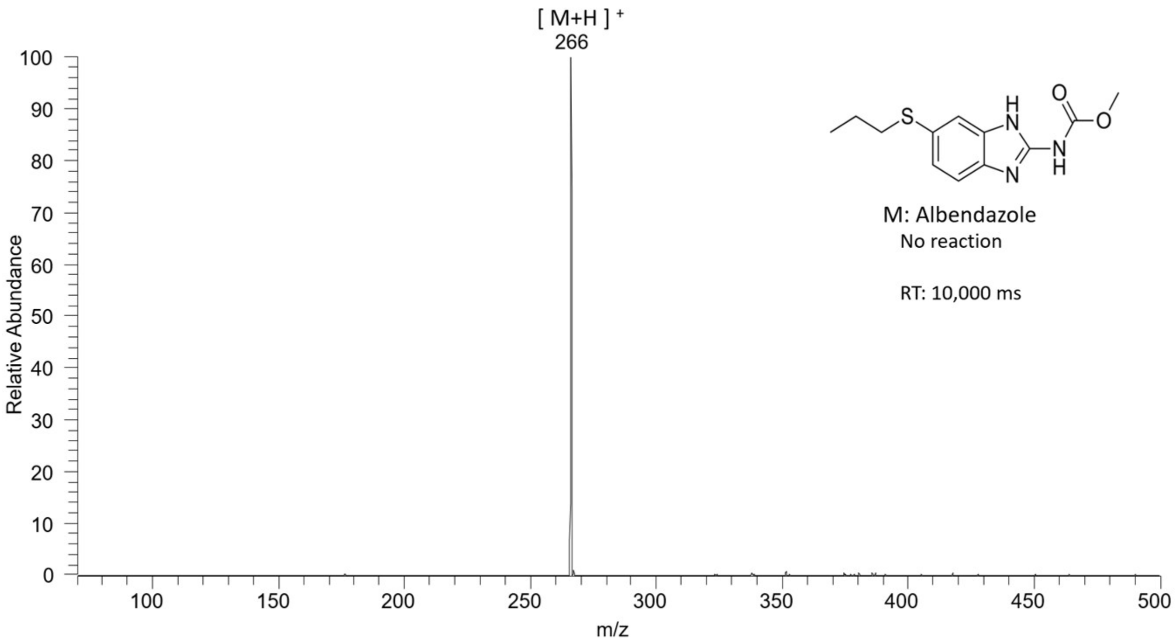

Figure S8. MS/MS spectrum measured after 10,000 ms reaction of protonated albendazole with MOP, indicating that a diagnostic addition product was not formed. No proton transfer product was observed, either.

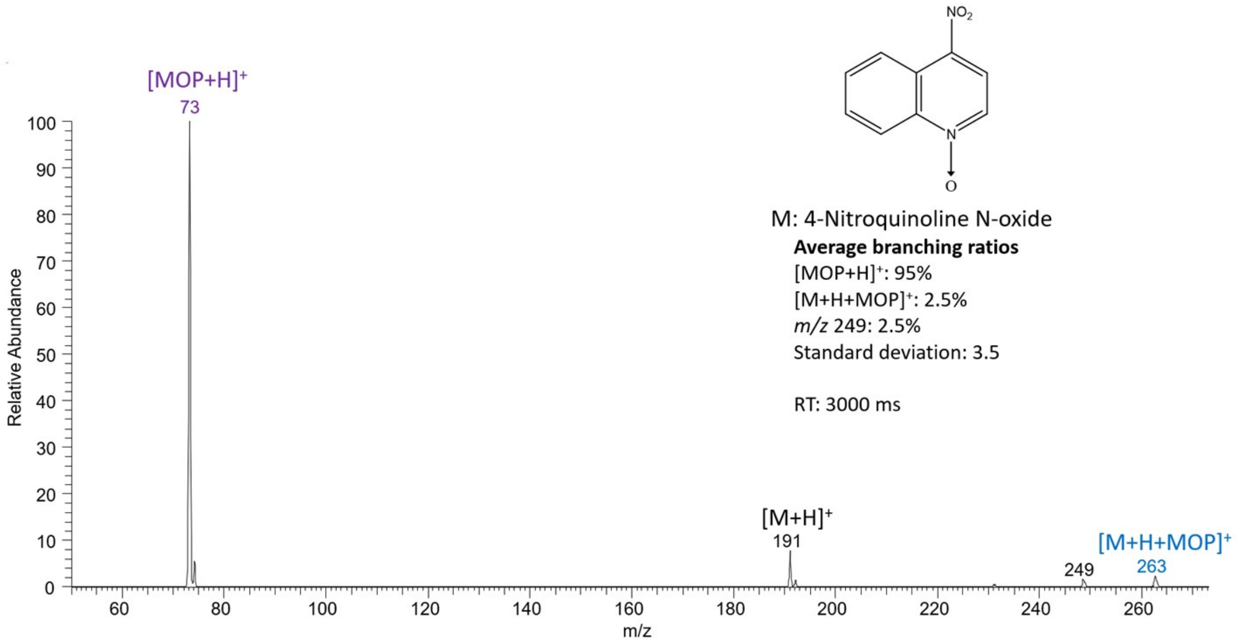

Figure S9. MS/MS spectrum measured after 3,000 ms reaction of protonated 4-nitroquinoline N-oxide with MOP. Although evidence of a diagnostic addition product is seen, the presence of a major proton transfer product indicates that this reaction is not suitable for diagnostic applications.

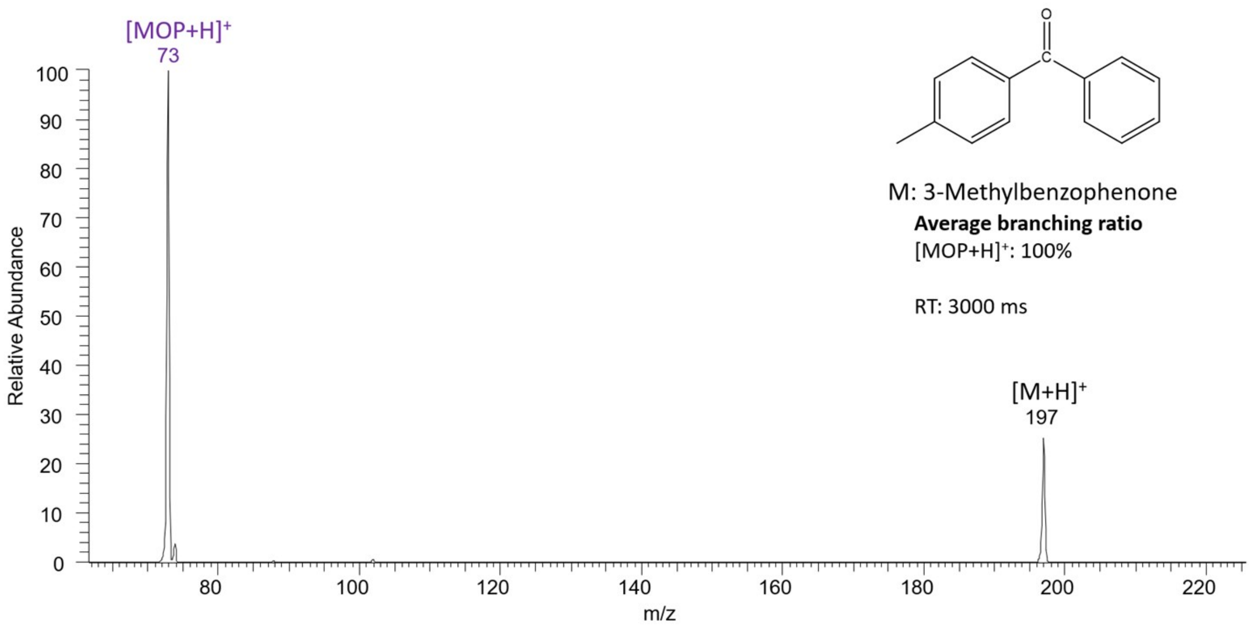

Figure S10. MS/MS spectrum measured after 3,000 ms reaction of protonated 3-methylbenzophenone with MOP, indicating that a diagnostic addition product was not formed. Instead, a proton transfer product was observed.

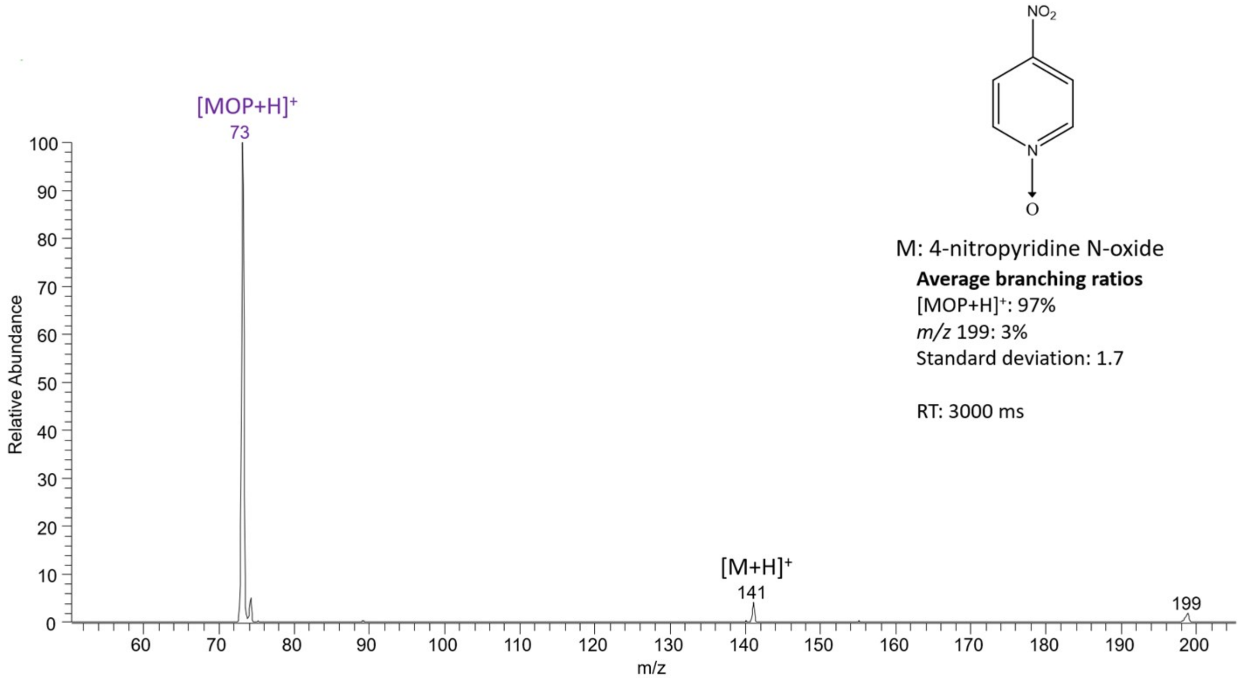

Figure S11. MS/MS spectrum measured after 3,000 ms reaction of protonated 4-nitropyridine N-oxide with MOP, indicating that a diagnostic addition product was not formed. Instead, a proton transfer product was observed.

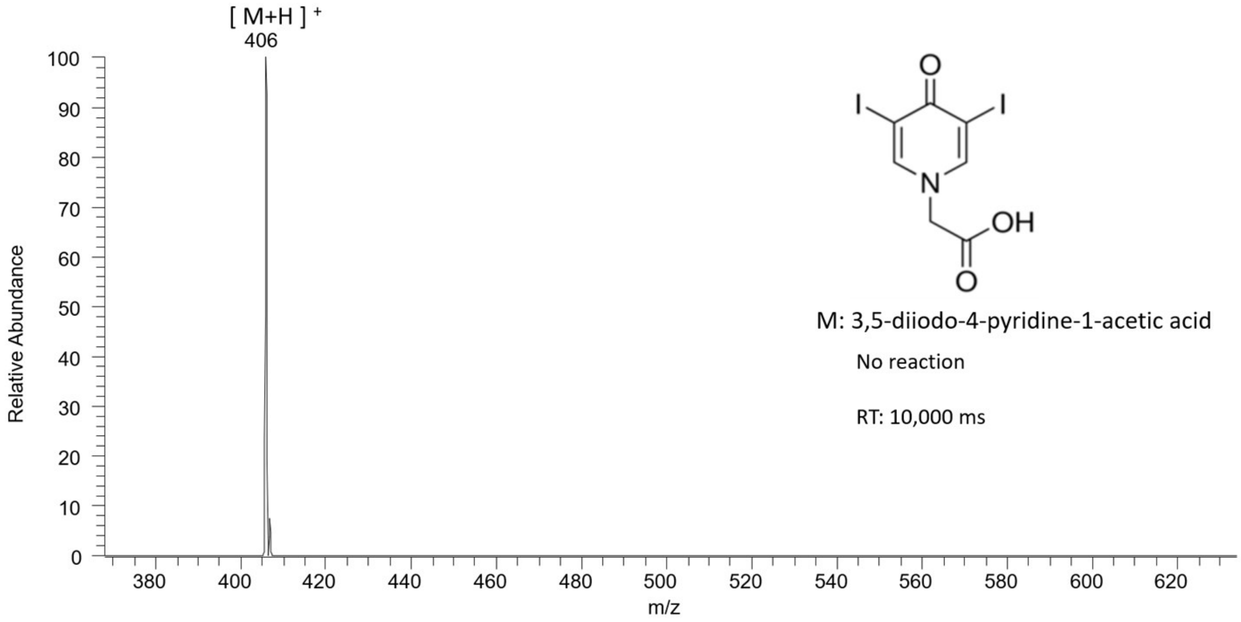

Figure S12. MS/MS spectrum measured after 10,000 ms reaction of protonated 3,5-diiodo-4-pyridine-1-acetic acid with MOP, indicating that a diagnostic addition product was not formed. No proton transfer product was observed, either.

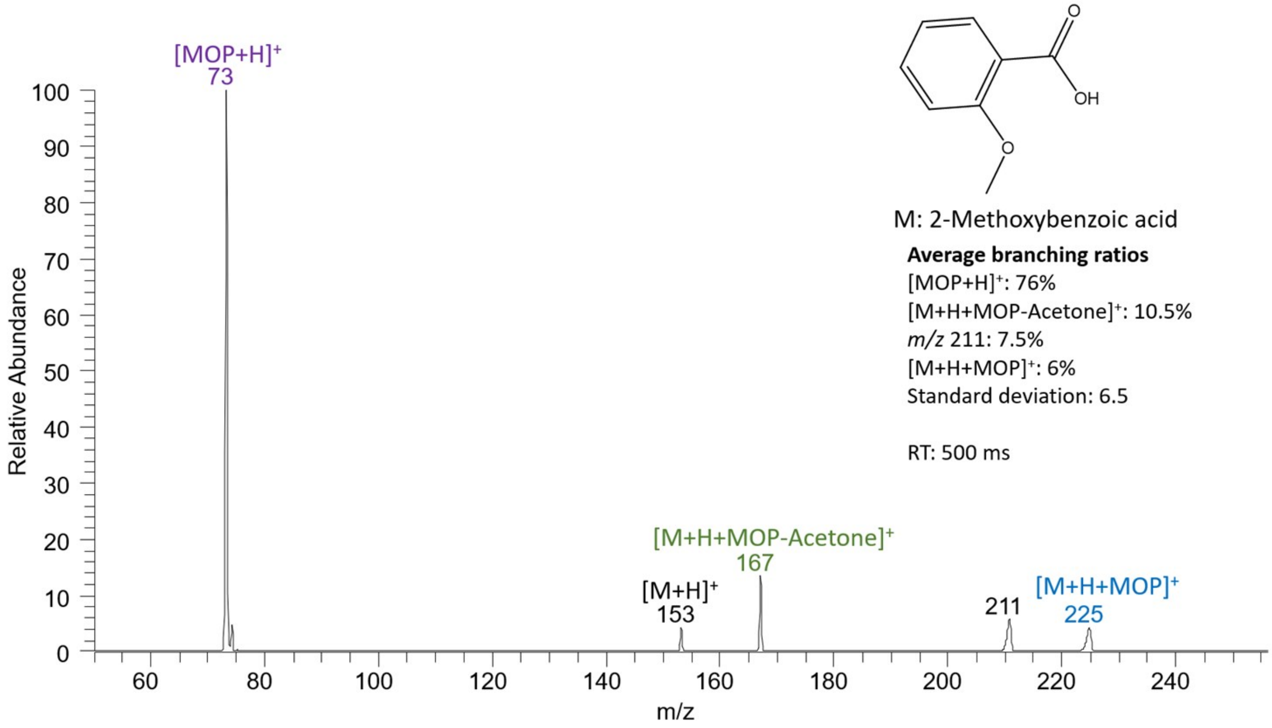

Figure S13. MS/MS spectrum measured after 500 ms reaction of protonated 2-methoxybenzoic acid with MOP. Although evidence of a diagnostic addition product is seen, the presence of a major proton transfer product indicates that this reaction is not suitable for diagnostic applications.

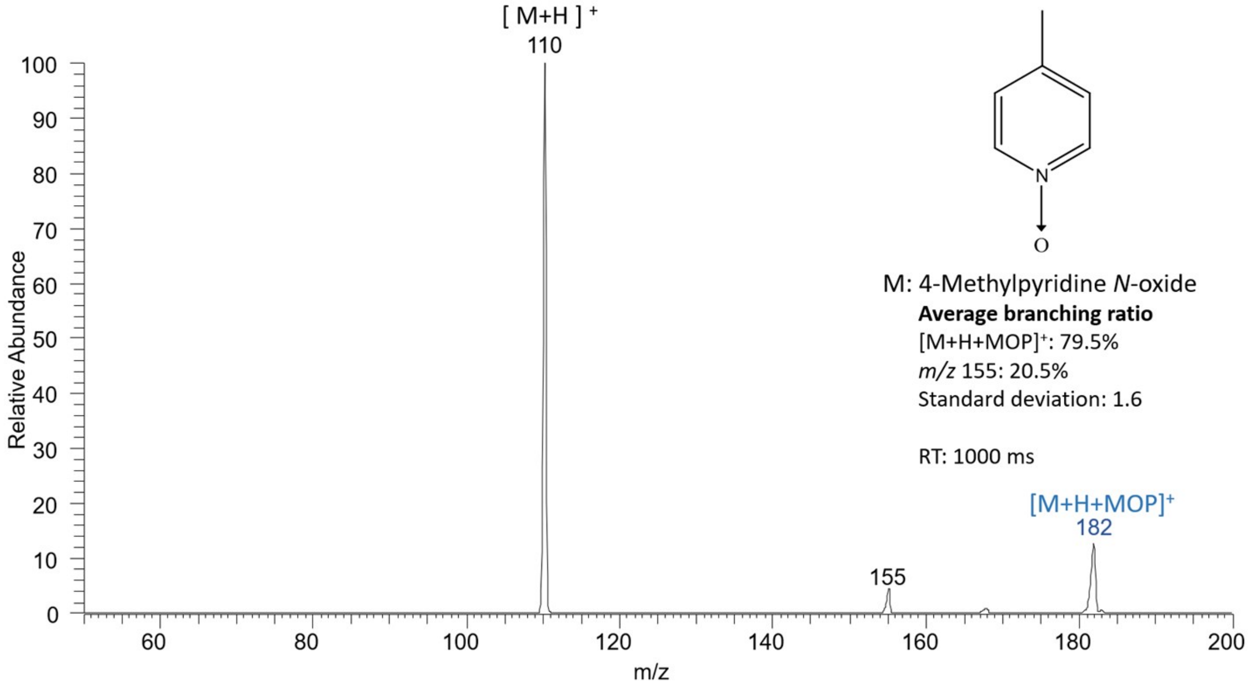

Figure S14. MS/MS spectrum measured after 1000 ms reaction of protonated 4-methylpyridine *N*-oxide with MOP, indicating the formation of a diagnostic addition product. No proton transfer product was observed.

The diagnostic product branching ratio is the fraction of product ions that have the mass of the analyte plus a proton plus the mass of MOP relative to all product ions generated. These values were measured at several reaction times and found to be constant with time as no secondary reactions took place. The average branching ratios were obtained by calculating the mean of the branching ratios of each product ion tested on different experimental days. The standard deviation of the most abundant product ion formed in each reaction tested on different experimental days were also obtained using the equation below.

$$\textit{Sample standard deviation} = \sqrt{\frac{1}{N-1}\sum_{i=1}^{N}(x_i - \bar{x})^2}$$

N: number of experimental days

x_i*: the observed branching ratio (most abundant product ion) of each day*

$\bar{x}$*: the mean value of the branching ratios (most abundant product ion) for all experimental days*
